# Degradative Capacity of Two Strains of *Rhodonia placenta*: From Phenotype to Genotype

**DOI:** 10.3389/fmicb.2020.01338

**Published:** 2020-06-18

**Authors:** Martina Kölle, Maria Augusta Crivelente Horta, Minou Nowrousian, Robin A. Ohm, J. Philipp Benz, Annica Pilgård

**Affiliations:** ^1^Chair of Wood Science, TUM School of Life Sciences Weihenstephan, Technical University of Munich, Munich, Germany; ^2^Professorship for Wood Bioprocesses, TUM School of Life Sciences Weihenstephan, Technical University of Munich, Freising, Germany; ^3^Department of Molecular and Cellular Botany, Ruhr University Bochum, Bochum, Germany; ^4^Department of Biology, Microbiology, Utrecht University, Utrecht, Netherlands; ^5^Institute of Advanced Study, Technical University of Munich, Garching, Germany; ^6^Biobased Materials, Bioeconomy, RISE Research Institutes of Sweden, Borås, Sweden

**Keywords:** *Rhodonia placenta*, *Postia placenta*, brown rot, genome comparison, standardized decay tests, wood degradation, hydrolytic enzymes

## Abstract

Brown rot fungi, such as *Rhodonia placenta* (previously *Postia placenta*), occur naturally in northern coniferous forest ecosystems and are known to be the most destructive group of decay fungi, degrading wood faster and more effectively than other wood-degrading organisms. It has been shown that brown rot fungi not only rely on enzymatic degradation of lignocellulose, but also use low molecular weight oxidative agents in a non-enzymatic degradation step prior to the enzymatic degradation. *R. placenta* is used in standardized decay tests in both Europe and North America. However, two different strains are employed (FPRL280 and MAD-698, respectively) for which differences in colonization-rate, mass loss, as well as in gene expression have been observed, limiting the comparability of results. To elucidate the divergence between both strains, we investigated the phenotypes in more detail and compared their genomes. Significant phenotypic differences were found between the two strains, and no fusion was possible. MAD-698 degraded scots pine more aggressively, had a more constant growth rate and produced mycelia faster than FPRL280. After sequencing the genome of FPRL280 and comparing it with the published MAD-698 genome we found 660,566 SNPs, resulting in 98.4% genome identity. Specific analysis of the carbohydrate-active enzymes, encoded by the genome (CAZome) identified differences in many families related to plant biomass degradation, including SNPs, indels, gaps or insertions within structural domains. Four genes belonging to the AA3_2 family could not be found in or amplified from FPRL280 gDNA, suggesting the absence of these genes. Differences in other CAZy encoding genes that could potentially affect the lignocellulolytic activity of the strains were also predicted by comparison of genome assemblies (e.g., GH2, GH3, GH5, GH10, GH16, GH78, GT2, GT15, and CBM13). Overall, these mutations help to explain the phenotypic differences observed between both strains as they could interfere with the enzymatic activities, substrate binding ability or protein folding. The investigation of the molecular reasons that make these two strains distinct contributes to the understanding of the development of this important brown rot reference species and will help to put the data obtained from standardized decay tests across the globe into a better biological context.

## Introduction

Brown rot fungi such as *Rhodonia placenta* (Fr.) *Niemelä, K.H. Larss. & Schigel* (previously *Postia placenta*) naturally occur in the northern forest ecosystems and are known to be the most destructive species, albeit representing only a small group within wood decay fungi (Zabel and Morrell, 1992; Goodell, 2003; Vanden Wymelenberg et al., 2010). It has been suggested that this is because brown rot fungi circumvent the lignin, leaving it behind in a highly modified state (Cowling, 1961; Kleman-Leyer et al., 1992; Suzuki et al., 2006; Schwarze, 2007; Arantes et al., 2011; Yelle et al., 2011; Schilling et al., 2015). Brown rot fungi do not only rely on enzymatic degradation of lignocellulose but use low molecular weight oxidative agents prior to the enzymatic degradation (Goodell et al., 1997; Arantes et al., 2012; Arantes and Goodell, 2014).

It is still not fully understood how the non-enzymatic oxidative degradation phase proceeds in detail. The currently most accepted theory is that brown rot fungi secrete oxalic acid, which diffuses into the lumen, where it sequesters Fe^3+^ as a chelator (Goodell et al., 1997; Eastwood et al., 2011; Arantes et al., 2012). Fe^2+^ is then formed through reduction by hydroquinones and other reducing agents, while additionally hydrogen peroxide (H_2_O_2_) is formed; likely through a reaction between hydroquinones and oxygen (Paszczynski et al., 1999; Jensen et al., 2001; Eastwood et al., 2011; Arantes et al., 2012; Melin et al., 2015). Hydroxyl radicals are then generated through the reaction of H_2_O_2_ and Fe^2+^ (Fenton reaction), causing cellulose and hemicellulose depolymerization and lignin modification (Fenton, 1894; Baldrian and Valášková, 2008; Arantes et al., 2012). Oligosaccharides, solubilized during this process, diffuse through the cell walls into the lumen, where they become accessible to cellulases and hemicellulases (Martinez et al., 2005; Goodell et al., 2017).

The number of genes encoding lignin-related enzymes is typically extremely reduced in the genomes of brown rot fungi. Most do not encode class II lignin-modifying peroxidases (AA2) (Riley et al., 2014) and laccase genes are either completely missing, as in *Gloeophyllum trabeum* (Floudas et al., 2012), or very limited in number in *R. placenta* (Martinez et al., 2009; Wei et al., 2010). Cellobiohydrolases, belonging to the GH families 6 and 7, attack cellulose and are often accompanied by a carbohydrate-binding module (mostly CBM1). In most brown rot fungi (including *Rhodonia placenta*) cellobiohydrolases are either absent or lacking a CBM1 domain (Lombard et al., 2013; Riley et al., 2014). Moreover cellobiose dehydrogenases (family AA3_1) are absent in the majority of brown rot fungi, and genes from cellulolytic families (GH5, GH12, GH44, GH45) are reduced. Still they are able to depolymerize and degrade polysaccharides from the wood cell wall (Martinez et al., 2009; Eastwood et al., 2011; Floudas et al., 2012; Riley et al., 2014). It is noteworthy that the number of enzymes active on hemicelluloses is not reduced to the same extent as cellulose-active enzymes (Martinez et al., 2009; Wei et al., 2010).

The dikaryotic strain MAD-698 of *Rhodonia placenta* was first sequenced by Martinez et al. (2009). Since then, there have been several studies on transcriptomics, proteomics and expression of single genes likely involved in wood decay (Vanden Wymelenberg et al., 2010; Ryu et al., 2011; Ringman et al., 2014; Alfredsen et al., 2016a; Zhang et al., 2016; Pilgård et al., 2017; Zhang and Schilling, 2017; Beck et al., 2018). The results of these studies imply that there is a time-wise and spatial separation between the non-enzymatic oxidative and the enzymatic degradation phase. Especially genes involved in the non-enzymatic oxidative degradation are significantly upregulated during early decay. Later decay stages are dominated by expression of GH family genes (Zhang et al., 2016). The transition from non-enzymatic oxidative to enzymatic degradation is triggered by the release of inducer molecules, particularly cellobiose (Zhang and Schilling, 2017). Investigations of the secretome seemed to confirm these trends (Presley et al., 2018). Findings by us and others (Ringman et al., 2014; Alfredsen et al., 2016a, b; Ringman et al., 2016; Pilgård et al., 2017; Kölle et al., 2019; Ringman et al., 2020), also imply that there might be additional mechanisms in the regulation of the non-enzymatic degradation beyond the cellobiose switch (Zhang and Schilling, 2017).

Since brown rot fungi can cause massive damage in a short period of time, wood products and wood protection systems need to be vigorously tested prior to permission for commercial use. This is performed according to national standardized tests (Cowling, 1961; Filley et al., 2002; Niemenmaa et al., 2007; Arantes et al., 2012; Arantes and Goodell, 2014). In standardized decay tests, *R. placenta* is used as a representative brown rot fungus. Two widely used strains of *R. placenta* are MAD-698 and FPRL280. MAD-698 is the recommended strain in the US American Wood-preservers’ Association Standard (AWPA E10-16, 2016), while FPRL280 is the recommended strain in the European standard EN 113 (CEN EN 113, 1996).

Differences in mass loss, colonization-rate, as well as in gene expression between the *R. placenta* MAD-698 and the FPRL280 strains have been observed (Thaler et al., 2012), leading to the assumption that there might be significant differences in either the genome, gene regulation, or in post-transcriptional mechanisms involved in the degradation process. Importantly, these observations raise fundamental concerns about the comparability of results obtained with these two strains.

While the corresponding monokaryotic strain to MAD-698, MAD-SB12, was sequenced more recently (Gaskell et al., 2017), the genome of the monokaryotic *R. placenta* strain FPRL280 has not been analyzed so far. To investigate the degradative capacity of the “European” *R. placenta* strain FPRL280 in comparison with the “American” *R. placenta* strain MAD-698/MAD-SB12, we thus sequenced its genome and performed parallel standardized decay tests, aiming to identify differences between the two strains that might help to explain the observed variances in phenotype. Comparisons of the genomes of different *Rhodonia* strains has to our knowledge not been performed before. In this study, our goal was to deliver solid genomic data on differences between MAD-SB12 and FPRL280 that can be used for further functional analysis. While contributing to a better understanding of the species’ lineage and hopefully providing a reference for future studies using either one of the two strains, the knowledge gained may also be of great interest for new biomass conversion technologies (Mester et al., 2004; Goodell et al., 2008; Schilling et al., 2012).

## Materials and Methods

### Strains

An isolate of *Rhodonia placenta* (Fr.) Niemelä, K.H. Larss. & Schigel (previously *Postia placenta*) FPRL280 was used in this study. MAD-698 is a dikaryotic strain (Martinez et al., 2009) and was used for phenotype tests and phenotype comparisons. MAD-SB12, a monokaryotic strain (Gaskell et al., 2017), was used for a fusion test, mapping of the FPRL280 sequence and for genotype comparisons. FPRL280 is a monokaryotic strain and was used for phenotype tests, sequencing as well as for genotype comparisons.

### Phenotype Analysis

#### Growth Test and Appearance

To investigate growth speed and growth behavior, 4% malt agar plates were inoculated with either *R. placenta* FPRL280 or *R. placenta* MAD-698. Measurement of the growth progress started on day two and the hyphal front was measured daily until the mycelium reached the edges of the plate (8 days). The diameter was measured in two directions, and a mean was calculated. Growth appearance of *R. placenta* strain FPRL280 and MAD-698 was observed and evaluated subjectively, when growing either on 4% malt agar dishes or on wood samples.

#### Mass Loss Test

The mass loss test was done according to the method presented by Bravery (Bravery, 1979). Sterile nets were placed on 4% malt agar petri dishes and inoculated with either *R. placenta* FPRL280 or *R. placenta* MAD-698. When the plates were completely overgrown, miniblock wood samples (10 mm × 5 mm × 30 mm) of scots pine (*Pinus sylvestris* L.) were placed on top of the nets. Samples were harvested after 1, 2, 3, 4, 5, 6, and 7 weeks of incubation at 22°C and 70% relative humidity, dried and weighted.

#### Fusion Test

Possible fusion between *R. placenta* strain FPRL280 and strains MAD-698 and MAD-SB12 was tested on 4% malt agar petri dishes.

### Genotype Analysis

#### Sequencing, Mapping, and *de novo* Assembly

*Rhodonia placenta* (Fr.) FPRL280 was cultivated on 4% malt agar plates for 14 days at room temperature (25°C). The DNA was extracted following the protocol by Traeger et al. (2013) (see Appendix). Library prep was done using the Illumina TruSeq PCR free kit (insert size 350 bp), according to the TruSeq DNA Sample Preparation Guide (Part #15036187, Rev.D, June 2015). The DNA libraries were paired-end (2 × 126) sequenced on an Illumina HiSeq2500 equipment. Paired-end reads were quality-trimmed to remove reads with undefined bases and trim reads from the 5′ and 3′ends until a base with phred-quality ≥ 10 was reached, with minimum length 60 bp.

The reads were mapped against the dikaryotic *R. placenta* MAD-698 genome, but the described comparison was done with the monokaryotic *R. placenta* MAD-SB12 genome^1^ (Gaskell et al., 2017) using Bowtie 2 (Langmead and Salzberg, 2012) version 2.2.6 for comparison, and SAMtools version 1.4 (Li et al., 2009) and BCFtools version 1.4 for variant calling. The reads were also *de novo* assembled using SPAdes (Bankevich et al., 2012) version 3.10. Gene prediction on SPAdes assembly was done with Maker (Cantarel et al., 2008). The following options were set in the CTL file of maker: protein2genome = 1, always_complete = 1. Maker was run with the [-RM_off] option, otherwise default parameters were used. The assembly was annotated based on the combined predicted proteins from the previously published assembly (Martinez et al., 2009; Gaskell et al., 2017). The predicted proteins from the *de novo* annotation were used for prediction of the 11,486 orthologous genes using reciprocal blast analysis and MAD-SB12 (Gaskell et al., 2017) as reference genome.

#### Annotation of CAZy-Encoding Genes

The public CAZy list determined by Gaskell et al. (2017) with 317 CAZy genes was used to find the corresponding CAZy genes in the *de novo* assembly of FPRL280. The CAZy gene sequences in coding sequences (CDSs) from MAD-SB12 were used to find the corresponding gene sequences within the entire set of CDSs from FPRL280, and only the best hits were selected, based on *e*-value, score and identity (Supplementary Data Sheet 1). A total of 298 genes were identified this way and another 16 sequences were when searching against the full *de novo* genome assembly. For four CAZy genes from MAD-SB12 no significant *e*-values could be retrieved by both methods, indicating that these genes do not exist in the FPRL280 *de novo* genome assembly (POSPLADRAFT_1141676, POSPLADRAFT_1141705, POSPLADRAFT_1042744, and POSPLADRAFT_1121407).

#### Validation of Genome Assembly

Polymerase chain reaction (PCR)-based Sanger sequencing of seven CAZy-encoding genes was performed to validate the differences found in the *de novo* assembled FPRL280 genome compared to MAD-SB12. Primers were designed based on the FPRL280 *de novo* assembly, and the sequences from the PCR product of the gDNA amplification were compared with the published sequence of MAD-SB12 (Supplementary Table 1 and Supplementary Figure 2). Sequences were compared using CLC Genomics Workbench 20.0 (QIAGEN, Aarhus, Denmark). For those genes which were not found in the FPRL280 *de novo* assembly, primers were designed based on MAD-SB12 sequence and used to probe for the corresponding genes in the gDNA of both strains.

The whole genome project has been deposited in the Sequence Read Archive (SRA) of NCBI under accession numbers PRJNA606481 and SAMN14091738 to the sample reads and to the assembly.

#### Phylogenetic Analysis

The species phylogeny was reconstructed using highly conserved gene products of the previously published genome annotations of the species indicated in Figure 5 (Martinez et al., 2009; Eastwood et al., 2011; Fernandez-Fueyo et al., 2012; Floudas et al., 2012; Olson et al., 2012; Suzuki et al., 2012; Tang et al., 2012; Binder et al., 2013; Ohm et al., 2014; Nagy et al., 2015; Miettinen et al., 2016; Wu et al., 2018; Casado López et al., 2019). BUSCO v2 (dataset “fungi_odb9”) was used to select 179 highly conserved proteins for the species phylogeny (Simão et al., 2015). These sequences were concatenated and aligned with MAFFT 7.307 (Katoh and Standley, 2013) and well-aligned regions were identified with Gblocks 0.91b (Talavera and Castresana, 2007). This resulted in 86,531 amino acid positions. RAxML version 8.1.16 was used for the phylogenetic tree reconstruction using the PROTGAMMAWAG similarity matrix and 100 bootstraps (Stamatakis, 2014). The phylogenetic tree was visualized and rooted on the outgroups *S. lacrymans* and *H. annosum* using Dendroscope (Huson and Scornavacca, 2012).

## Results

### Phenotypic Comparison Between FPRL280 and MAD-698

**FIGURE 1:**
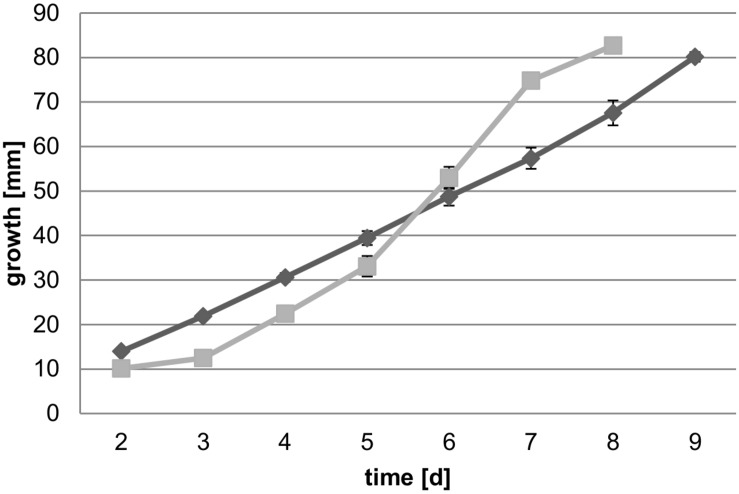
Growth speed of both strains on 4% malt agar plates over 9 days (*n* = 5). Average daily growth rates of FPRL280 (gray squares) and MAD-698 (black diamonds) are shown with standard deviations in the graph and imply that MAD is starting growth earlier and grows more evenly than FPRL.

When incubated on malt extract agar plates, MAD-698 was found to grow faster during the first 5 days, whereas FPRL280 started its growth delayed, but accelerated and caught up with MAD-698 between days 5 and 6 (Figure 1).

**FIGURE 2:**
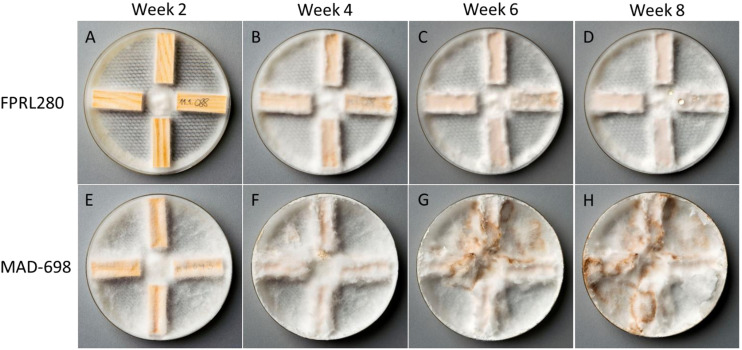
Wood degradation test to observe appearance of both strains (FPRL280 and MAD-698) when growing on miniblock wood samples. **(A–D)** Show growth of FPRL280 after 2–8 weeks. **(E–H)** Show growth of MAD-698 after 2–8 weeks. Clear differences can be seen in these pictures, not only in appearance of the mycelium, but also in growth speed and mycelium production.

Differences were also observed in the general appearance, when growing on petri dishes with 4% malt agar. MAD-698 grew more multidirectional than FPRL280, which also grew closer to the surface of the medium, directed to the edges of the plates (Figures 2, 4). The mycelium of MAD-698 produced more aerial hyphae growing upwards, giving the mycelium a more voluminous appearance (Figures 2, 4). When growing on wood samples, there were also differences between the two fungi. MAD-698 clearly produced more mycelium during the 8-week degradation test (Figure 2).

**FIGURE 3:**
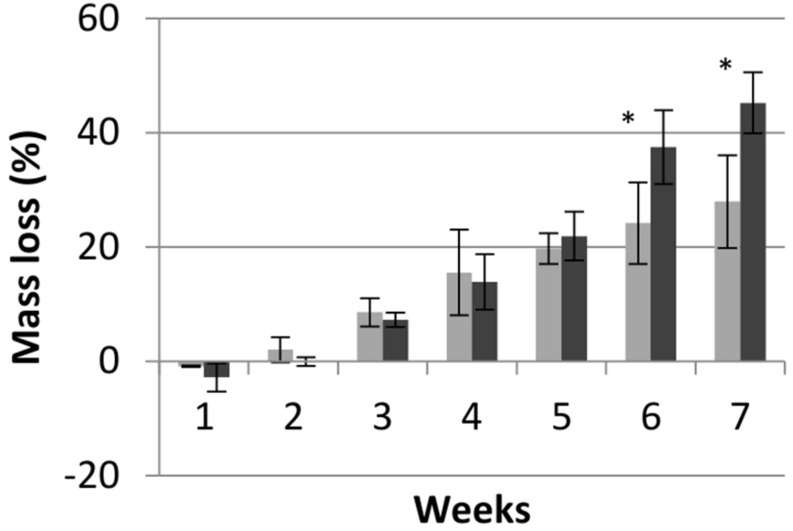
Mass loss of pine wood samples over time when incubated with FPRL280 (gray) and MAD-698 (black). Data show means of biological (*n* = 4) and technical replicates (*n* = 8) with standard deviations, showing that MAD is causing a higher mass loss than FPRL after 6 weeks. *T*-test was used to determine the significance, with *p* < 0.05. “^∗^” means significant differenced between the strains (*p* < 0.05).

To determine differences in wood degradation capacity a mass loss test on wooden miniblock samples was performed over 7 weeks (Figure 3). Despite showing a delayed growth phenotype on malt agar, FPRL280 started degrading the wood earlier than MAD-698 but then slowed down. After 6 and 7 weeks, mass loss by MAD-698 was significantly stronger, suggesting that MAD-698 is degrading the wood in a more aggressive manner than FPRL280 in the long term.

#### Fusion Test

**FIGURE 4:**
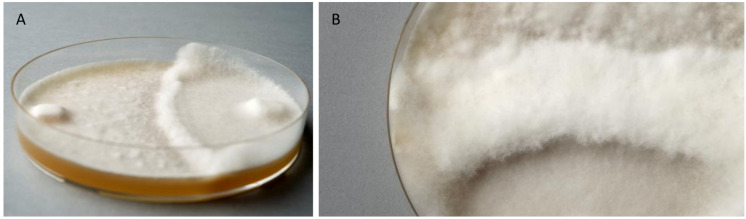
Fusion test of the two *Rhodonia placenta* strains growing on one plate. **(A)** FPRL280 (left side) and MAD-698 (right side), 2 weeks after incubation, clearly showing the formation of a barrier, which is not overgrown. **(B)** The barrier got thicker and neither of the fungi overgrew it.

To test whether FPRL280 and MAD-698 are able to fuse, the strains were confronted with each other on the same plate. A border was immediately built up between the two *R. placenta* strains when the mycelia met, which both fungi did not overgrow (Figure 4A). The same was observed for FPRL280 and the corresponding monokaryotic strain of MAD, MAD-SB12 (Supplementary Figure 3). Further days of investigation showed that even on the edges of the plates, the fungi grew upwards, but not over the demarcation line. After several weeks, there was a broad band of mycelium looking like a wall built up between the two strains (Figure 4B).

### Sequencing of FPRL280 and Comparison With MAD-SB12

Next, we sequenced the genome of *R. placenta* strain FPRL280. Since the genome assembly of strain MAD-698 is much more fragmented and gap-rich than the more recent MAD-SB12 assembly, mapping rates of the genome reads of FPRL280 were much lower, reducing the quality of downstream variant analysis (Table 1). We therefore decided to focus our genomic comparison on the monokaryotic strain MAD-SB12 and a *de novo* assembly. *In* the *de novo* genome assembly, 12,997 genes were predicted for FPRL280, which is about the same number as reported for MAD-SB12 (12,541), 11,486 of these being putative orthologs (Supplementary Data Sheet 1 and Table 2).

**TABLE 1:** Comparison of genome assemblies for MAD-698 (Martinez et al., 2009), MAD-SB12 (Gaskell et al., 2017), and FPRL280.

	MAD-698 assembly	SB12 assembly	FPRL280
Assembly size (Mb)	90.9	42.5	36.3
No. of scaffolds	1243	549	1848
No. of gaps within scaffolds	10184	897	192
Total size of gaps (Mb)	21.9	2.6	0.007
% of scaffold length in gaps	24.1	6.1	0.02
Mapping rate of FPRL280 reads (%)	64	80	NA

**TABLE 2:** Assembly and annotation features for *R. placenta* FPRL280.

Feature	Value
Genome assembly size (Mbp)	39
No. of contigs	3,158
No. of scaffolds	2,948
No. of scaffolds ≥ 1000 bp	1,848
Scaffold N50	74 kb
Gene models	12,997

**FIGURE 5:**
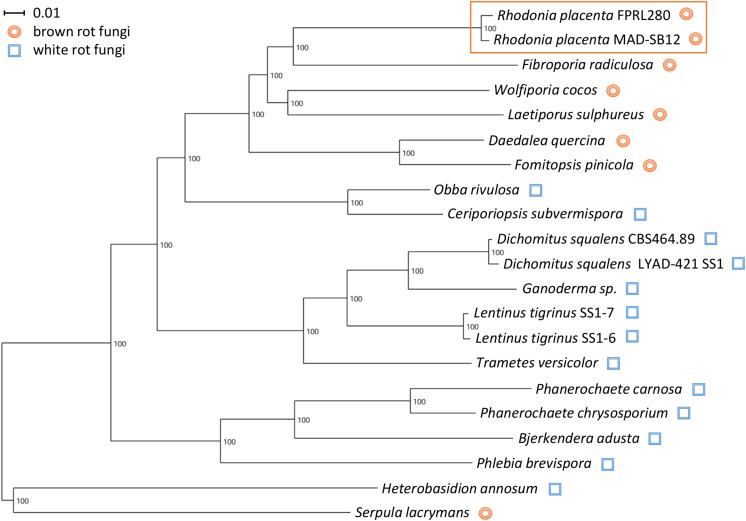
Phylogenetic tree of members of the order Polyporales based on 179 highly conserved proteins. *S. lacrymans* (order Russulales) and *H. annosum* (order Boletales) were used as outgroup to root the tree. The strains of *R. placenta* cluster closely together, as expected for closely related strains (orange box).

A comparison with the MAD-SB12 genome showed an overall identity of 98.4% with 660,566 SNPs and 25,837 indels, translating roughly into one difference per 60 basepairs on average (Table 3 and Supplementary Data Sheet 2). In addition, precise classification is presented in Supplementary Data Sheet 2, showing the affected genes, changes in amino acid sequences, the position and the kind of variance. An overview on how the SNPs are distributed over introns, exons, UTRs, or if they occur in intergenic regions can be found in Supplementary Figure 4. A total of 686,402 variants were detected within the whole genome (introns included), 16,299 variants (2.37%) residing in CAZy genes. A phylogenetic tree based on 179 highly conserved gene products shows that both *R. placenta* strains are closely related. The evolutionary distance between them is similar to the distance between strains of *Lentinus tigrinus* and *Dichomitus squalens* (Figure 5).

**TABLE 3:** Overview of the variant analysis between FPRL280 and MAD-SB12 genomes.

	All variants	Variants in CAZy genes
Total no. of variants	686,403(100%)	16,299(2.37%)
SNPs	660,566(96.24%)	15,888(2.40%)
Indels	25,837(3.76%)	411(1.61%)

**FIGURE 6:**
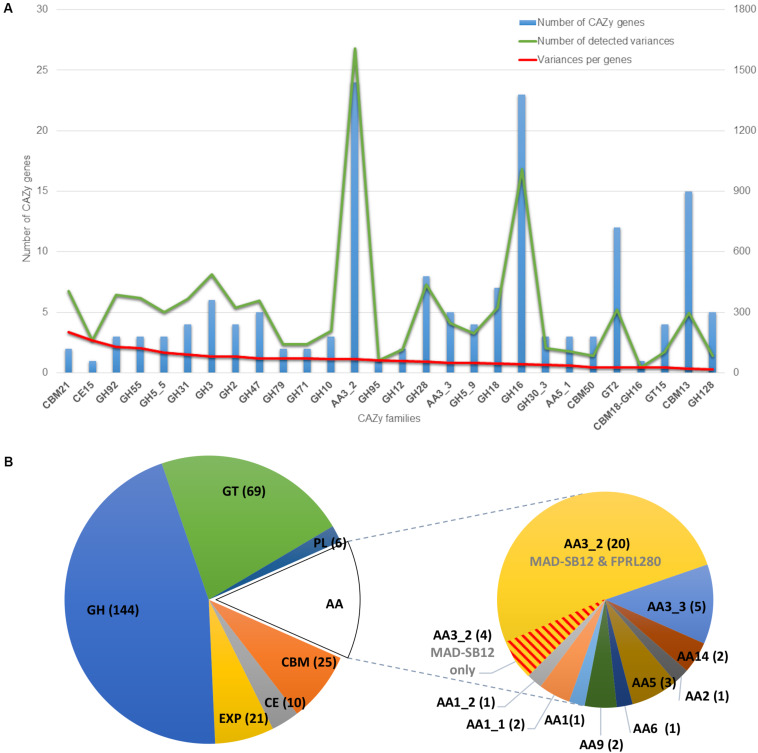
CAZy classification. **(A)** Variant analysis of CAZy genes of FPRL280 compared to MAD-SB12 (only CDS). The graphic only shows CAZy families mentioned in the text, a graphic with results for all CAZy families can be found as Supplementary Figure 1. The blue bar shows the number of CAZy genes with variants within the CAZy subfamilies. The green line shows the number of all variants occurring in the CAZy subfamilies. The red line shows the variances within the CAZy subfamilies normalized to their size. **(B)** Entire classification of CAZy-encoding genes in both genomes, highlighting class AA, with the subfamily AA3_2, in which many variants were found and four genes were missing in FPRL280 vs. MAD-SB12 (red line pattern). The piecharts contain CAZy families AA, CBM, CE, GH, GT, and PL, as well as expansins (EXP).

Next, we compared all detected variants between FPRL280 and MAD-SB12 vs. variants that are present in predicted CAZyme genes (Table 4 and Supplementary Data Sheet 3). Table 4 presents results for the variant analysis between FPRL280 and MAD-SB12 as well as the number of coding sequences (CDS) and CAZy genes being affected. Within the CDS, we found a total of 10,027 genes with amino acid changes (AACs), of which 69% had between one and ten AACs and 1.2% having more than 50 AACs. Of all CAZy-encoding genes, almost all (92.43%) had at least one AAC, including 46 AAs, 136 GHs and 24 CBMs. Additionally, 17 genes with deletions, 30 with insertions and 20 frame shifts through indels were detected within the CAZy genes. Thirty-three CAZy genes were identified that had variants in consensus splice sites (VCSSs), the largest group here being the AA3_2 subfamily with eight genes and the GH5_5 subfamily with two genes. Overall, insertions appeared in 9.46% and deletions in 5.36% of all CAZy genes. Summarized results of the variant analysis, considering the sizes of the CAZy subfamilies, can be seen in Figures 6A,B. For an overview of variants in all CAZy families see Supplementary Figure 1.

**TABLE 4:** Detailed variant analysis and number of affected genes with corresponding percentage according to total number of CDS genes (all variants) or total number of CAZy genes (variants in CAZy genes).

	All variants		Variants in CAZy genes	
	Total No. of variants	No. of genes	Total No. of variants	No. of genes
AACs (in CDS)	115,302	10,027(79.95%)	3,301	293(92.43%)
Deletions (in CDS)	1,177	929(7.41%)	20	17(5.36%)
Insertions (in CDS)	2,123	1,233(9.83%)	48	30(9.48%)
Frameshifts through indels	1,499	954(7.61%)	25	20(7.89%)
VCSSs (in CDS)	1,194	984(7.85%)	38	33(10.41%)

**FIGURE 7:**
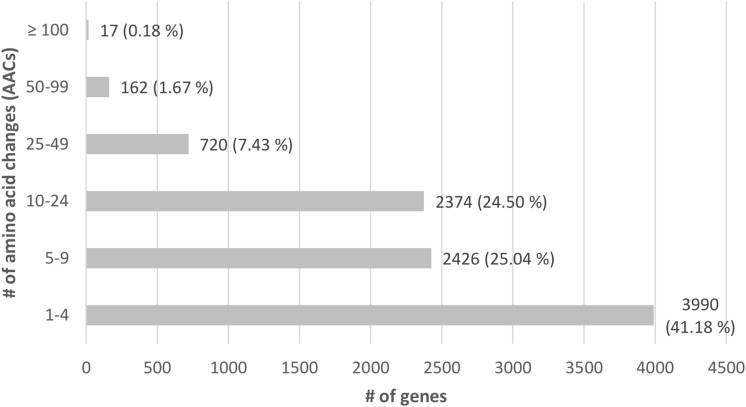
All genes with amino acid changes (AACs) after comparing MAD-SB12 and FPRL280, according to amount of AACs (*y*-axis) and number of genes with corresponding percentage (*x*-axis).

Of all genes having AACs, 162 genes (1.67%) had ≥ 50 instances and 17 (0.18%) even ≥ 100 (Figure 7). Genes with 50 and more AACs were looked at in more detail, which showed that 23 of these were affected over more than 10% of their length, 107 genes were affected between 5 and 10% and 29 genes ≤ 5%. The GO terms regarding the annotated molecular functions of the genes were investigated and grouped. Proteins with associated functions that might contribute the observed phenotypic differences were present in this group. For example, 15 genes had RNA-, DNA or nucleic acid binding functions, based on UniProt search of the respective *R. placenta* MAD-SB12 homologs, or WD-repeat regions involved in a wide range of protein-protein interactions in signal transduction processes, cell division, RNA processing and so on (Yu et al., 2000; Smith, 2008; Stirnimann et al., 2010). The list furthermore was enriched for F-box-domain containing proteins (further referred to as F-box proteins; eight of a total of 164 predicted in the genome; *p*-value < 5 × 10^–3^), and showed an overrepresentation of proteins with kinase function (15 out of a predicted total of 398; *p*-value < 5 × 10^–4^). Further alignment of the protein sequences showed that the domains of two F-box proteins, four protein kinases as well as two WD-repeats regions are heavily affected (Table 5). Screening all variants showed four genes which had additional variants besides AACs: FPRL280_424_1 (POSPLADRAFT_1046092) had three VCSSs as well as a one-base insertion leading to a frameshift; FPRL280_272 (POSPLADRAFT_1065282) had an additional VCSS, FPRL280_188_6 (POSPLADRAFT_1035374) showed one five-base, one one-base and one two-base deletion, leading to frameshifts, three one-base and two four-base insertions, also leading to frameshifts as well as one VCSS. Further FPRL280_104_5 (POSPLADRAFT_1046501) had additionally two VCSSs and a three-base insertion, not leading to a frameshift.

**TABLE 5:** Genes with ≥ 50 AACs, which were looked at in more detail due to their predicted effect on the phenotype.

Protein	Affected genes	Avg. degree of affection (% of the gene length)	No. of AACs (Avg.)
F-Box protein	8	10.24	69.25
Kinases	15	6.13	62.4
WD repeats	3	7.75	107.7
DNA binding	7	4.87	56.43
Nucleic acid binding	4	5.92	71
RNA binding	4	5.01	66

**The table includes the percentage of bases affected, relative to the total protein length and the number of AACs, as well as the protein.**

### Identification and Validation of Variants in CAZyme-Encoding Genes

**FIGURE 8:**
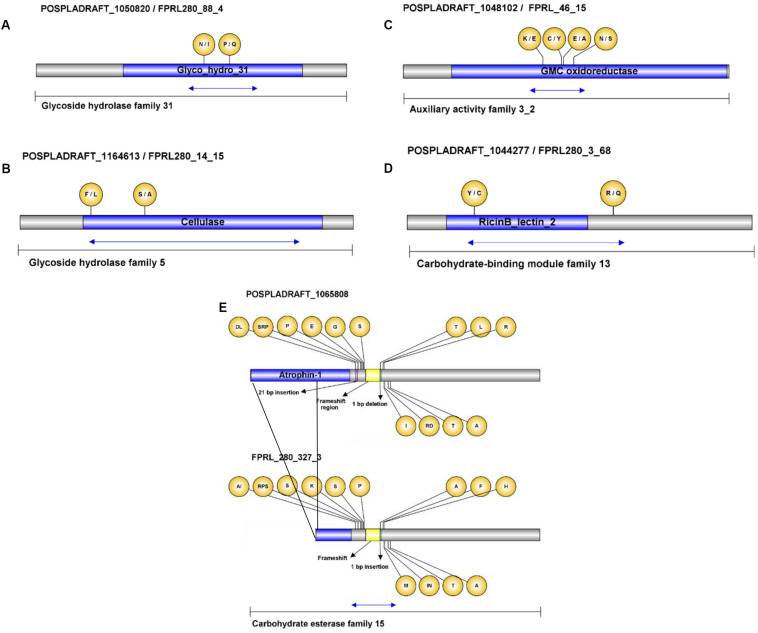
Visualization of variances and amino acid changes in selected CAZy genes. The differences were confirmed by gDNA amplification of FPRL280 followed by comparison with the published MAD-SB12 gene sequence and the FPRL280 *de novo* genome assembly. The amino acid changes are shown in the bubbles, MAD-SB12/FPRL280 **(A–D)**, and as indicated **(E)**. The blue arrows indicate the partial sequences verified by Sanger sequencing. The black lines in **(E)** represent the region that is missing in the FPRL280 homolog.

Genomic regions of selected genes from GH31, AA3_2, GH16, CBM18, GH5, and CE15 family members were amplified and sequenced to verify the observed variances in the FPRL280 genome (Supplementary Data Sheet 4). All mentioned genes are consecutively numbered and shown in Table 6 with corresponding IDs for MAD-SB12 and FPRL280, as well as the predicted effects of the variants. The regions tested by PCR amplification are indicated in Figure 8.

**TABLE 6:** All genes mentioned in the results are numbered with both IDs (MAD-SB12 and FPRL280), the CAZy family these genes belong to and the predicted effects of variances, causing AACs, VCSSs or frameshifts.

ID MAD-SB12 “POSPLADRAFT_”	ID FPRL280 “FPRL280_”	CAZy family	Predicted effect
1050820	88_4	GH31	AAC (positions 480 and 553) in coding protein XP_024333100.1, mutations, leading to frameshifts
1164613	14_15	GH5_5	AAC (positions 72 and 126) in protein XP_024344095.1, VCSS
1048102	46_15	AA3_2	4 AACs (positions 272, 308, 312, and 332) in protein XM_024479440.1
1044277	3_68	CBM13	AAC (positions 30 and 90) in protein XP_024341629.1
1065808	327_3	CE15	Mutations, leading to frameshifts
1046599	156_2	GH95	Mutations, leading to frameshifts, VCSS
1057601	47_16	GH30_3	Mutations, leading to frameshifts, VCSS
1066962	238_7	GH71	Mutations, leading to frameshifts, deletion (4 bases) in EC 3.2.1.59
1138061	218_2	GH47	Mutations, leading to frameshifts, insertion (8 bases) and deletion (4 bases) in enzyme-encoding region (EC 3.2.1.113); VCSS
1155254	318_2	GH79	Mutations, leading to frameshifts
1168110	279_6	GH18	Mutations, leading to frameshifts
1181115	252_4	GH16	Mutations, leading to frameshifts
1064814	264_7	GH10	VCSS
1050186	142_19	GH12	VCSS
1183855	4_136	GH128	VCSS
1142572	640_1	GH16	VCSS
1043339	NODE_261	GH2	VCSS
1049546	348_3	GH28	VCSS
1174812	49_4	GH3	VCSS
1042537	14_27	GH5_5	VCSS
1181612	432_1	GH5_9	VCSS
1131418	269_3	GH55	VCSS
1043572	294_2	GH92	VCSS

Within the GH31 domain of gene FPRL280_88_4, as well as in the cellulose domain of gene FPRL280_14_15 several SNPs and AACs could be confirmed (Figures 8A,B). Thirteen SNPs were verified to exist in gene FPRL280_46_15, a predicted oxidoreductase (AA3_2; PFAM00732/PFAM05199) with putative cellobiose dehydrogenase or alcohol oxidase activity (EC 1.1.99.18 or EC 1.1.3.13), in a small region of the domain (Figure 7C) and the RicinB_lectin domain (PFAM14200) of gene FPRL280_3_68 (Figure 7D). High number of variants were furthermore detected in gene FPRL280_327_3, with predicted 4-O-methyl-glucuronoyl methylesterase activity (EC 3.1.1) in a region close to the functional domain (Figure 7E), predict to allow the frameshift.

In addition to the selected genes shown in Figure 7, it was possible to confirm SNPs (including two AACs) within CBM13 domain-containing gene, and further AACs in CBM18, CBM21, CBM48, and CBM50 genes (Table 5 and Supplementary Data Sheet 3). Other CBM13-encoding genes (FPRL280_235_10 and FPRL280_174_14) presented variances in consensus splice sites and insertions/deletions leading to putative frameshifts. Mutations leading to frameshifts were also detected in genes belonging to the CAZy families CBM21 and CBM50, as well as members of GH95, GH31, GH30_3, GH71, GH47, GH79, GH18, and GH16 (Table 5 and Supplementary Data Sheet 3). Fourteen GH families were affected by mutations within predicted consensus splice sites: GH10; GH12; GH128; GH16; GH2; GH28; GH3; GH30_3; GH47; GH5_5; GH5_9; GH55; GH92; GH95 (Table 5).

Four genes (POSPLADRAFT_1141676, POSPLADRAFT_11 41705, POSPLADRAFT_1042744, and POSPLADRAFT_112 1407) present in the MAD-SB12 genome could not be detected in or amplified from the FPRL280 genome. All four genes belong to the CAZy subfamily AA3_2 (Supplementary Figure 2).

## Discussion

In the present study, we performed a direct comparison of both phenotype and genotype of two commonly used strains of *R. placenta*, FPRL280 and MAD-698 (or MAD-SB12, respectively). We chose to directly compare the phenotypes of MAD-698 (a dikaryon) and FPRL280 (which, suggested by the genome assembly and by absence of microscopically visible clamps, is a monokaryon), since these are the two strains being used in laboratories in the US and Europe for standardized decay test of wood products (AWPA E10-16, 2016). However, for the genome comparison with FPRL280, we chose to use the monokaryotic MAD-SB12 genome. The genome size determined for FPRL280 was much closer to that of MAD-SB12 and mapping efficiencies were substantially higher (Table 1). Since the genome of MAD-SB12 was isolated from a basidiospore of the fruiting dikaryon MAD-698, the genome is as close to the formely reported genome of MAD-698 as possible (Gaskell et al., 2017).

Differences in growth behavior, wood decomposition effectiveness and mycelial appearance were observed and quantified as well as vegetative incompatibility between the strains. Since it has been shown that monokaryons and dikaryons of *Trametes versicolor* have similar decay rate, combative ability and ligninolytic enzymes production (Hiscox et al., 2010), we assume that this does not account as a major influencing factor in *R. placenta* as well. While the observed vegetative incompatibility might have been caused by the different nuclear status nevertheless, this behavior was also seen for FPRL280 and MAD-SB12 (the respective monokaryon; Supplementary Figure 3), which indicates that the incompatibility depends on genetic differences rather than on differences in nuclear status.

One to two percent of the gene products in eukaryotic organisms belong to the GT family (Lairson et al., 2008), which are necessary, among others, for the biosynthesis of the fungal cell wall (Klutts et al., 2006). GTs are enzymes that utilize an activated sugar substrate, containing a phosphate leaving group (Lairson et al., 2008) and catalyze the transfer of sugar moieties from donor molecules to specific acceptors, leading to the formation of glycosidic bonds (Campbell et al., 1997; Coutinho et al., 2003). Five putative GT genes were found having AACs in FPRL280. Potentially therefore, these mutations could be part of the reason why the MAD-698 mycelia have a different appearance than FPRL280. However, since the GTs include 110 families and we don’t know which effects the VCSS have on the enzymes (Lairson et al., 2008), this remains speculative until further analysis.

The differences in phenotype make the comparison interesting both in terms of genomic research and industrial applications. Mutations in important genes are one explanation for the differences seen in phenotype between the two species, but also regulatory mechanisms are likely having a powerful impact. The two genomes are closely related with a total identity of 98.4% and as confirmed by a phylogenetic analysis (Figure 5). The results of the variant analysis nevertheless showed a high number of potentially impactful differences between the two strains. For example, amino acid changes (AAC) were found in 92% of all predicted CAZy genes, as well as deletions in 5% and insertions in 9%. Moreover, variants in consensus splicing sites (VCSS) were found in 10% of all CAZys. Overall, a higher percentage of AACs and VCSSs were found in CAZy genes compared to the entire complement of CDSs (Table 2), often affecting structural domains. We also found that four genes belonging to the CAZy family AA3_2 could not be detected in the FPRL280 genome, potentially explaining part of the observed differences regarding wood decay rates (see also below).

Brown rot fungi possess less GHs than white-rot fungi, but they seem to compensate this by secreting higher amounts of their remaining GHs (Presley et al., 2018). Brown rot fungi generally lack processive cellobiohydrolases and instead rely more on endoglucanases, which are thought to cleave cellulose randomly (Eriksson et al., 1990; Vanden Wymelenberg et al., 2010). Putative endo-acting cellulases belong to the CAZy families GH5 and GH12 (Ryu et al., 2011; Floudas et al., 2012; Lombard et al., 2013). Zhang et al. (2016) found only three endoglucanases (GH5 and GH12) and one putative endoglucanase (GH12) in *R. placenta*. We found mutations in the well-characterized endo-1,4-β-D-glucanase PpCel5A (FPRL280_14_15; Ppl1| 115648; POSPLADRAFT_1164613; XP_024344095) belonging to the GH5 family and including the endo-1,4-β-glucanase FPRL280_142_19 (Ppl1| 52805/Ppl1| 112669; POSPLADRAFT_1050186; XP_024333913) belonging to the GH12 family in FPRL280. Mutations were also found in several putative hemicellulases, such as the β-mannosidase FPRL280_NODE_261 (Ppl1| 57564; POSPLADRAFT_1043339; XP_024342514), the α-1,2-mannosidase FPRL280_294_2 (Ppl1| 62385; POSPLADRAFT_1043572; XP_024342867) and the β-xylosidase FPRL280_9_74 (Ppl1| 127469; POSPLADRAFT_1069652; XP_024341044). In addition, we found that several putative β-glucosidase-encoding genes in FPRL280 have mutations (Supplementary Data Sheet 3).

Hemicellulases can be found in several GH families and are often co-operating to break down complex hemicelluloses (Ryu et al., 2011; Brigham et al., 2018). Moreover, β-glucosidases are relatively non-specific in brown rot fungi (Herr et al., 1978; Valášková and Baldrian, 2006). It could be hypothesized that the detected mutations in hemicellulases and β-glucosidases will not affect the decay capability of FPRL280 as much as mutations in the endoglucanases since several β-glucosidases have been found in *R. placenta* (Martinez et al., 2009) and only three to four endoglucanases (Zhang et al., 2016). The sequence variances seen in GH family enzymes, in particularly the endoglucanases, could thus be one part of a possible explanation to the overall lower decomposition rate by FPRL280 compared to MAD-698, since these enzymes are responsible for the depolymerization of the structural carbohydrates in the wood cell wall (Ryu et al., 2011; Floudas et al., 2012; Lombard et al., 2013).

During early stages of brown rot decay, CEs are required for hemicellulose removal, for example to remove acetyl groups from xylan in hardwoods (Cowling, 1961; Puls, 1997). Presley et al. (2018) found an increased spectrum of genes attributed to CEs in brown rot secretomes, particularly in early stages of decay. In one protein putatively belonging to the CE15 family (POSPLADRAFT_1065808/FPRL280_327_2; XP_024339306) we found a high number of AACs in the FPRL280 ortholog compared to MAD-SB12 and VCSSs. These mutations might affect the hemicellulose depolymerization in FPRL280 negatively.

As mentioned above, brown rot fungi rely on non-enzymatic break-down of lignocellulose using low molecular weight compounds, such as H_2_O_2_, Fe^2+^ and oxalate for Fenton chemistry (Goodell et al., 1997; Arantes et al., 2009; Arantes and Goodell, 2014), for an efficient lignocellulose degradation. The genomes of brown rot fungi suggest the presence of a number of AA enzymes that are known to generate H_2_O_2_. Among these are AA3 GMC oxidoreductases and AA5 copper radical oxidases (Floudas et al., 2012; Levasseur et al., 2013). AA3 are a family of flavoenzymes that oxidize aliphatic alcohols, aryl alcohols and mono- and disaccharides. This oxidation is coupled with the reduction of a variety of electron acceptors, including O_2_ (resulting in the formation of H_2_O_2_), quinones and other enzymes. Enzymes belonging to the AA3_2 subfamily include two closely related FAD-dependent enzymes, aryl-alcohol oxidase and glucose-1-oxidase (Levasseur et al., 2013; Sützl et al., 2018). Flavoproteins, such as GMC oxidoreductases, form the base of a wide array of biological processes, for example removal of radicals, which contribute to oxidative stress adaptation. The CAZy group with the highest number of variances in FPRL280 was the AA3_2 subfamily. We found eight putative genes with VCSS in the FPRL280 genome. In addition to this, four AA3_2-encoding genes were missing in the FPRL280 genome. Taking into account the suggested importance of hydroquinones for the H_2_O_2_ production, mutations in AA3_2 proteins, or the absence of entire proteins, could thus have severe effects on the degradation capacity of FPRL280. Enzymes belonging to the AA5 family are copper radical oxidases and are known to be a major constituent of the secretome of several brown rot fungi (Kersten and Cullen, 2014). AA5s oxidize a variety of substrates resulting in the production of H_2_O_2_ via the reduction of O_2_ (Jensen et al., 2002). The AA5 family includes two subfamilies, AA5_1 containing characterized glyoxal oxidase and AA5_2 containing galactose oxidase, raffinose oxidase and alcohol oxidase enzymes (Ito et al., 1991). In a copper radical oxidase (Ppl1| 56703 POSPLADRAFT_1046361/FPRL280_259_1; XP_024339806) belonging to the subfamily AA5_2, we found VCSS. The fact that four AA3_2 genes are missing in FPRL280 and that parts of the domains are missing in enzymes in both AA3_2 and AA5_2, in addition to high numbers of gaps and point mutations, also in VCSS, might lead to changes with possible effects on the protein functions. Since members of the AA3 family do not directly act on polymeric constituents of lignocellulosic material, but support degradation by reducing low-molecular weight components, with one of the main products being H_2_O_2_ (Sützl et al., 2018), changes in AA3_2 genes could affect this support substantially. The effect of this might be a less effective Fenton reaction in FPRL280 compared to MAD-698, which could explain the phenotypical findings that MAD-698 is more potent during prolonged decay, producing higher mass losses than FPRL280. Brown rot fungi have most likely a fine-tuned complex network of gene products working together to deliver radicals via the Fenton reaction. Perhaps even small changes in these gene products could lead to measurable effects. Whether this is indeed the case, however, needs to be verified by future experiments.

A proportionally large part of the variances found in FPRL280 are located in regulatory genes which could have a huge impact on the phenotype far beyond the mere differences of the two genomes.

Nucleic acid binding proteins, for example, are important factors involved in gene expression (Latchman, 1997). Zinc-finger proteins belonging to this group can act as transcription factors and form one of the largest families if transcriptional regulators in eukaryotes, with an enormous functional diversity (Miller et al., 1985; MacPherson et al., 2006). One representative (POSPLADRAFT_1155119/FPRL280_66_2) was found to be affected by more than 50 AACs in this study. Mutations in genes coding for these proteins are therefore likely to contribute to the overall complexity of the phenotypes of both strains, since their functional differences will affect entire downstream regulons.

Kinases, mediating phosphorylation reactions of proteins and other cellular constituents, are another example of important regulatory proteins involved in many signaling cascades. Within the pool of proteins found to have > 50 AACs between FPRL280 and MAD-SB12, kinases were found to be overrepresented (*p*-value < 5 × 10^–4^) indicating that the function of several signaling pathways might be affected. However, the diversity of kinases is immense, and future efforts are necessary to identify exactly which pathways these are.

F-box motifs, also found to be overrepresented (*p*-value < 5 × 10^–3^), function as a site of protein-protein interaction and the respective proteins are key factors involved in protein ubiquination proteasomal degradation (Jonkers and Rep, 2009). As part of the Skp, Cullin, F-box containing complex (SCF complex; a multi-protein E3 ubiquitin ligase), F-box proteins have several target proteins, which suggests that mutations may also have a pleiotropic effect on the phenotype. In filamentous fungi, F-box proteins can be involved in several cellular processes, including control of the cell division cycle, sugar sensing, mitochondrial connectivity, and control of the circadian clock (Jonkers and Rep, 2009). The proteolytic function of the ubiquitin-proteasome system is furthermore important for virulence regulation in pathogenic fungi (Liu and Xue, 2011) and the variations in F-box domains in some proteins in FPRL280 might thus contribute to the lower virulence seen in FPRL280 compared to MAD-698.

Besides AACs, frame shift mutations and VCSSs can potentially have even more drastic effects on the amino acid sequences of proteins. However, for most CAZy genes we looked at in more detail, there variances did not affect the conserved functional domains. Moreover, splicing can be better observed by a transcriptional analysis. This is currently ongoing, including a gene expression regulation analysis, and will be part of another manuscript.

We limited the scope of this paper to a comparative genome study, since we wanted to highlight the phenotypic differences between the two *R. placenta* strains and the underlying genomes. A careful genomics analysis is very important, as it forms the basis for further research. However, genome analysis can clearly explain the differences between the strains only partially, and functional genomics studies, including transcription, translation, gene regulation and protein-protein-interactions, need to be included in the future for a more profound comparison.

## Conclusion

The initial reason for comparing the two strains was to gain insight into the genetic differences between two economically relevant strains of *R. placenta* (FPRL280 and MAD-698) showing different phenotypes. The specific mutations discussed in this paper might contribute to these observed differences as they are found in relevant domains of many potentially important genes especially regulatory genes, and thus might affect the function of the respective proteins. However, with 98.4% overall identity, the genomic variances cannot explain all observations. Differences in regulatory mechanisms (signaling cascades etc.) are likely also present and impactful, and therefore need to be further investigated.

The results from this paper show the importance of a united strain selection of decay fungi in standardized decay tests. Two strains of one species can behave differently even though the genomes appear similar. The investigation of the reasons that make these two strains distinct is useful for the understanding of the degradation mechanisms employed by brown rot fungi and the development of this important brown rot reference species.

## Data Availability Statement

The datasets generated for this study can be found in the NCBI databank under BioProject number PRJNA606481, and BioSample SAMN14091738.

## Author Contributions

AP, MK, JB, and MH initiated and designed the research. MK, MN, RO, and MH performed the analyses. AP, MK, JB, and MH co-wrote the manuscript with support of MN and RO. All authors were included in the interpretation of the data, read and approved the final manuscript.

## Conflict of Interest

The authors declare that the research was conducted in the absence of any commercial or financial relationships that could be construed as a potential conflict of interest.

## Footnotes

1 https://mycocosm.jgi.doe.gov/PosplRSB12_1/PosplRSB12_1.home.html
