# Supplementary material for: Degradative Capacity of Two Strains of Rhodonia placenta: From Phenotype to Genotype
Source: Front Microbiol. 2020 Jun 18;11:1338. doi: 10.3389/fmicb.2020.01338 (PMC7314958; doi:10.3389/fmicb.2020.01338)
Supplement: Supplementary file 4 [file Data_Sheet_4.PDF]

POSPLADRAFT_1050820
FPRL 280 _88_4
GH31 - POINT MUTATION POSITION 1493, 1515, 1691, 1791, 1806, 1857 CDs
AMINO ACID CHANGE POSITION 480, 553

Postia placenta MAD-698-R-SB12 glycoside hydrolase family 31 protein (POSPLADRAFT_1050820), partial mRNA

NCBI Reference Sequence: XM_024480074.1

GenBank FASTA

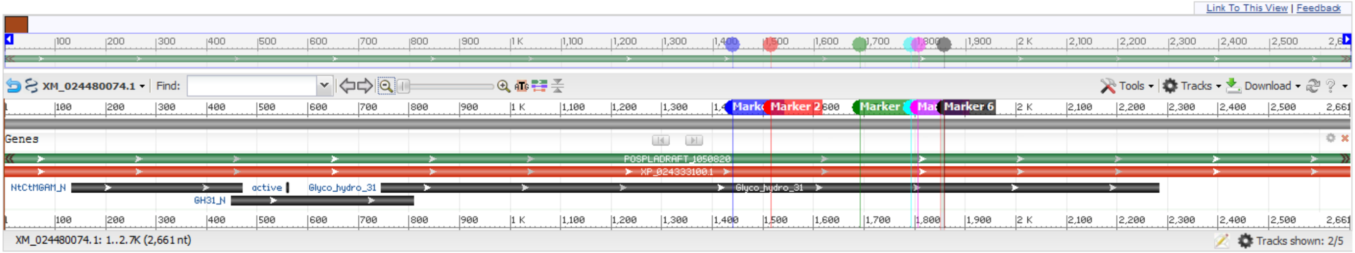

glycoside hydrolase family 31 protein [Postia placenta MAD-698-R-SB12]

NCBI Reference Sequence: XP_024333100.1

GenPept Identical Proteins FASTA

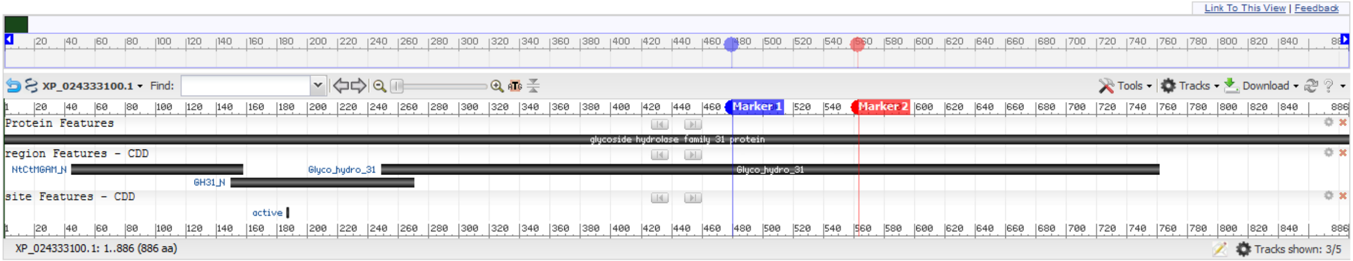

POSPLADRAFT_1050820
Glyco_hydro_31
1,340 1,360 1,380 1,400
XM_024480074 selection TGCACCTACC CATGTACGGA TCCATTCCAG CAAGCTGCCG AACAAGATCT TCCACCGCCG CGGACGACGA TCCCACCTGA 80
Translation ORF/CDS C T Y P C T D P F Q Q A A E Q D L P P P R T T I P P D

88_4 selection_CDS TGCACCTACC CGTGTACGGA TCCATTCCAG CAAGCTGCCG AACAAGATCT TCCACCGCCG CGGACGACGA TCCCACCTGA 80
Translation ORF/CDS C T Y P C T D P F Q Q A A E Q D L P P P R T T I P P D

EF30714023_EF30714023 TGCACCTACC CGTGTACGGA TCCATTCCAG CAAGCTGCCG AACAAGATCT TCCACCGCCG CGGACGACGA TCCCACCTGA 80
Translation ORF/CDS C T Y P C T D P F Q Q A A E Q D L P P P R T T I P P D
Consensus TGCACCTACC CGTGTACGGA TCCATTCCAG CAAGCTGCCG AACAAGATCT TCCACCGCCG CGGACGACGA TCCCACCTGA
Conservation
Sequence logo

POSPLADRAFT_1050820
Glyco_hydro_31
1,420 1,440 1,460 1,480
XM_024480074 selection CCCGAACGCG CCCATTTTCG TGAACTCAAC CGATTCTAAT GTCGTTTTGT CGCTGCAGAA GAGACAAAGC GATAGCGGCG 160
Translation ORF/CDS P N A P I F V N S T D S N V V L S L Q K R Q S D S G

88_4 selection_CDS CCCGAACGCG CCCATTTTCG TGAACTCAAC CGATTCTATT GTCGTTTTGT CGCTGCAGAA GAGACAAAGC GATAGCGGCG 160
Translation ORF/CDS P N A P I F V N S T D S I V V L S L Q K R Q S D S G

EF30714023_EF30714023 CCCGAACGCG CCCATTTTCG TGAACTCAAC CGATTCTATT GTCGTTTTGT CGCTGCAGAA GAGACAAAGC GATAGCGGCG 160
Translation ORF/CDS P N A P I F V N S T D S I V V L S L Q K R Q S D S G
Consensus CCCGAACGCG CCCATTTTCG TGAACTCAAC CGATTCTATT GTCGTTTTGT CGCTGCAGAA GAGACAAAGC GATAGCGGCG
Conservation
Sequence logo

POSPLADRAFT_1050820
Glyco_hydro_31
1,500 1,520 1,540 1,560
XM_024480074 selection AGGACGTTCT AAACCCTCCT TACGCCATTG ACAATGCTGC CGGAGCGTTG TCAAATAGGA CTTCATATAC GAATGCAGTA 240
Translation ORF/CDS E D V L N P P Y A I D N A A G A L S N R T S Y T N A V

88_4 selection_CDS AGGACGTTCT AAACCCTCCT TACGCCATTG ACAACGCTGC CGGAGCGTTG TCAAATAGGA CTTCATATAC GAATGCAGTA 240
Translation ORF/CDS E D V L N P P Y A I D N A A G A L S N R T S Y T N A V

EF30714023_EF30714023 AGGACGTTCT AAACCCTCCT TACGCCATTG ACAACGCTGC CGGAGCGTTG TCAAATAGGA CTTCATATAC GAATGCAGTA 240
Translation ORF/CDS E D V L N P P Y A I D N A A G A L S N R T S Y T N A V
Consensus AGGACGTTCT AAACCCTCCT TACGCCATTG ACAACGCTGC CGGAGCGTTG TCAAATAGGA CTTCATATAC GAATGCAGTA
Conservation
Sequence logo

POSPLADRAFT_1050820
Glyco_hydro_31
1,580 1,600 1,620 1,640
XM_024480074 selection CACGCGAACG GCCTGCTCGA CTATGACACC AACCTGTTCG GGACGATGAT GTCCACTGTG ACGCATTACG CCATGCTAGC 320
Translation ORF/CDS H A N G L L D Y D T N L F G T M M S T V T H Y A M L A

88_4 selection_CDS CACGCGAACG GCCTGCTCGA CTATGACACC AACCTGTTCG GGACGATGAT GTCCACTGTG ACGCATTACG CCATGCTAGC 320
Translation ORF/CDS H A N G L L D Y D T N L F G T M M S T V T H Y A M L A

EF30714023_EF30714023 CACGCGAACG GCCTGCTCGA CTATGACACC AACCTGTTCG GGACGATGAT GTCCACTGTG ACGCATTACG CCATGCTAGC 320
Translation ORF/CDS H A N G L L D Y D T N L F G T M M S T V T H Y A M L A
Consensus CACGCGAACG GCCTGCTCGA CTATGACACC AACCTGTTCG GGACGATGAT GTCCACTGTG ACGCATTACG CCATGCTAGC
Conservation
Sequence logo

POSPLADRAFT_1050820
Glyco_hydro_31
1,660 1,680 1,700 1,720
XM_024480074 selection TCGCCGACCA GGCCTCCGGA CGCTCGTGGT CACTCGCTCG ACGCCGAAGT GGCTCGGCGA CAACATGGCT GACTGGGAAC 400
Translation ORF/CDS R R P G L R T L V V T R S T P K W L G D N M A D W E

88_4 selection_CDS TCGCCGACCA GGCCTCCGGA CGCTCGTGGT CACTCGCTCG ACGCAGAAGT GGCTCGGCGA CAACATGGCT GACTGGGAAC 400
Translation ORF/CDS R R P G L R T L V V T R S T Q K W L G D N M A D W E

EF30714023_EF30714023 TCGCCGACCA GGCCTCCGGA CGCTCGTGGT CACTCGCTCG ACGCAGAAGT GGCTCGGCGA CAACATGGCT GACTGGGAAC 400
Translation ORF/CDS R R P G L R T L V V T R S T Q K W L G D N M A D W E
Consensus TCGCCGACCA GGCCTCCGGA CGCTCGTGGT CACTCGCTCG ACGCAGAAGT GGCTCGGCGA CAACATGGCT GACTGGGAAC
Conservation
Sequence logo

POSPLADRAFT_1050820
Glyco_hydro_31
1,740 1,760 1,780 1,800
XM_024480074 selection ACTACCGAAA CTCGATATCG GGAATCTTGA GCATGGCGAG CGTGTTCCAC GTGCCGATGG TGGGCGCAGA TATCTGCGGG 480
Translation ORF/CDS H Y R N S I S G I L S M A S V F H V P M V G A D I C G

88_4 selection_CDS ACTACCGAAA CTCGATATCG GGAATCTTGA GCATGGCGAG CGTGTTCCAC GTGCCGATGG TGGGTGCAGA TATCTGCGGA 480
Translation ORF/CDS H Y R N S I S G I L S M A S V F H V P M V G A D I C G

EF30714023_EF30714023 ACTACCGAAA CTCGATATCG GGAATCTTGA GCATGGCGAG CGTGTTCCAC GTGCCGATGG TGGGTGCAGA TATCTGCGGA 480
Translation ORF/CDS H Y R N S I S G I L S M A S V F H V P M V G A D I C G
Consensus ACTACCGAAA CTCGATATCG GGAATCTTGA GCATGGCGAG CGTGTTCCAC GTGCCGATGG TGGGTGCAGA TATCTGCGGA
Conservation
Sequence logo

POSPLADRAFT_1050820
Glyco_hydro_31
1,820 1,840 1,860
XM_024480074 selection TACGGGGACA CTACGGAGAC GCTATGTGCA AGATGGGCTA TGCTCGGAGC TTTCTATCCA TTCATGCGCG AT 552
Translation ORF/CDS Y G D T T E T L C A R W A M L G A F Y P F M R D

88_4 selection_CDS TACGGGGACA CTACGGAGAC GCTATGTGCA AGATGGGCTA TGCTCGGGGC TTTCTATCCA TTCATGCGCG AT 552
Translation ORF/CDS Y G D T T E T L C A R W A M L G A F Y P F M R D

EF30714023_EF30714023 TACGGGGACA CTACGGAGAC GCTATGTGCA AGATGGGCTA TGCTCGGGGC TTTCTATCCA TTCATGCGCG AT 552
Translation ORF/CDS Y G D T T E T L C A R W A M L G A F Y P F M R D
Consensus TACGGGGACA CTACGGAGAC GCTATGTGCA AGATGGGCTA TGCTCGGGGC TTTCTATCCA TTCATGCGCG AT
Conservation
Sequence logo

POSPLADRAFT_1044277
FPRL 280 _3_68
CBM13 - POINT MUTATION POSITION 77,89,214,264,269 CDs
AMINO ACID CHANGE POSITION 30, 90

Postia placenta MAD-698-R-SB12 carbohydrate-binding module family 13 protein (POSPLADRAFT_1044277), partial mRNA

NCBI Reference Sequence: XM_024478507.1
GenBank FASTA

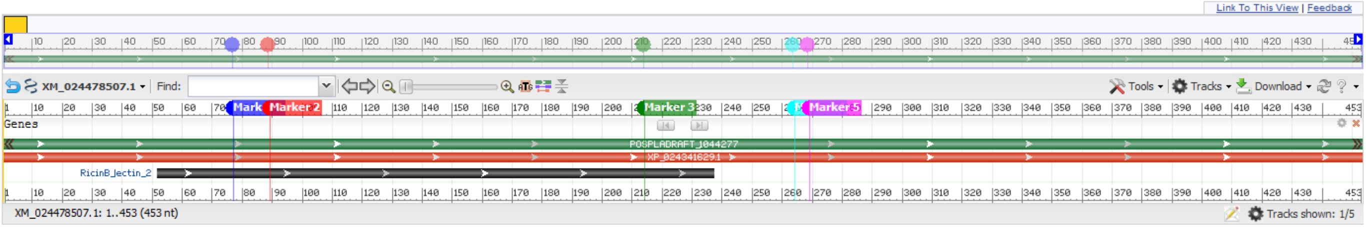

carbohydrate-binding module family 13 protein [Postia placenta MAD-698-R-SB12]

NCBI Reference Sequence: XP_024341629.1
GenPept Identical Proteins FASTA

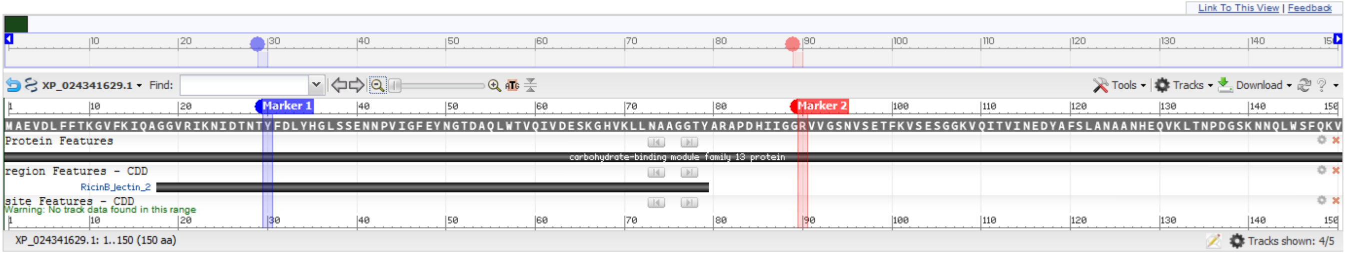

```
RicinB_lectin_2
POSPLADRAFT_1044277                          20                   40                   60
XM_024478507_1044277 selection  ATCAAGAACA TCGACACGAA CACCTACTTC GACTTGTACC ACGGCTTGAG TTCCGAGAAC AACCCCGTCA 70
Translation ORF/CDS              I  K  N   I  D  T  N   T  Y  F   D  L  Y   H  G  L  S   S  E  N   N  P  V

3_68 selection                  ATCAAGAACA TCGATACGAA CACCTGCTTC GACTTGTACC ACGGCTTGAG TTCCGAGAAC AACCCCGTCA 70
Translation ORF/CDS              I  K  N   I  D  T  N   T  C  F   D  L  Y   H  G  L  S   S  E  N   N  P  V

EF31080765_EF31080765           ATCAAGAACA TCGATACGAA CACCTGCTTC GACTTGTACC ACGGCTTGAG TTCCGAGAAC AACCCCGTCA 70
Translation ORF/CDS              I  K  N   I  D  T  N   T  C  F   D  L  Y   H  G  L  S   S  E  N   N  P  V
Consensus                       ATCAAGAACA TCGATACGAA CACCTGCTTC GACTTGTACC ACGGCTTGAG TTCCGAGAAC AACCCCGTCA
Conservation (100% / 0%)
Sequence logo (2.0bits / 0.0bits)
                                ATCAAGAACA TCGATACGAA CACCTGCTTC GACTTGTACC ACGGCTTGAG TTCCGAGAAC AACCCCGTCA

RicinB_lectin_2
POSPLADRAFT_1044277        80                   100                  120                  140
XM_024478507_1044277 selection  TCGGATTTGA GTATAACGGG ACCGATGCCC AGCTGTGGAC TGTCCAGATA GTCGATGAGA GCAAGGGACA 140
Translation ORF/CDS             I  G  F  E   Y  N  G   T  D  A   Q  L  W  T   V  Q  I   V  D  E   S  K  G  H

3_68 selection                  TCGGATTTGA GTATAACGGG ACCGATGCCC AGCTGTGGAC TGTCCAGATA GTCGATGAGA GCAAGGGACA 140
Translation ORF/CDS             I  G  F  E   Y  N  G   T  D  A   Q  L  W  T   V  Q  I   V  D  E   S  K  G  H

EF31080765_EF31080765           TCGGATTTGA GTATAACGGG ACCGATGCCC AGCTGTGGAC TGTCCAGATA GTCGATGAGA GCAAGGGACA 140
Translation ORF/CDS             I  G  F  E   Y  N  G   T  D  A   Q  L  W  T   V  Q  I   V  D  E   S  K  G  H
Consensus                       TCGGATTTGA GTATAACGGG ACCGATGCCC AGCTGTGGAC TGTCCAGATA GTCGATGAGA GCAAGGGACA
Conservation (100% / 0%)
Sequence logo (2.0bits / 0.0bits)
                                TCGGATTTGA GTATAACGGG ACCGATGCCC AGCTGTGGAC TGTCCAGATA GTCGATGAGA GCAAGGGACA

RicinB_lectin_2
POSPLADRAFT_1044277                          160                  180                  200
XM_024478507_1044277 selection  TGTCAAGCTC CTGAATGCCG CTGGCGGGAC CTATGCCCGT GCTCCCGACC ACATCATTGG TGGGCGAGTT 210
Translation ORF/CDS               V  K  L   L  N  A   A  G  G  T   Y  A  R   A  P  D   H  I  I  G   G  R  V

3_68 selection                  TGTCAAGCTC TTGAATGCCG CTGGCGGGAC CTATGCCCGT GCTCCCGACC ACATCATTGG CGGGCAAGTT 210
Translation ORF/CDS               V  K  L   L  N  A   A  G  G  T   Y  A  R   A  P  D   H  I  I  G   G  Q  V

EF31080765_EF31080765           TGTCAAGCTC TTGAATGCCG CTGGCGGGAC CTATGCCCGT GCTCCCGACC ACATCATTGG CGGGCAAGTT 210
Translation ORF/CDS               V  K  L   L  N  A   A  G  G  T   Y  A  R   A  P  D   H  I  I  G   G  Q  V
```

POSPLADRAFT_1048102

FPRL 280 _46_15

AA3_2 - POINT MUTATION POSITION 814, 816, 819, 843, 846, 849, 923, 933, 935, 951, 975, 978, 994 CDS

AMINO ACID CHANGE POSITION 272, 308, 312, 332 PROTEIN

Postia placenta MAD-698-R-SB12 hypothetical protein (POSPLADRAFT_1048102), partial mRNA

NCBI Reference Sequence: XM_024479440.1

GenBank FASTA

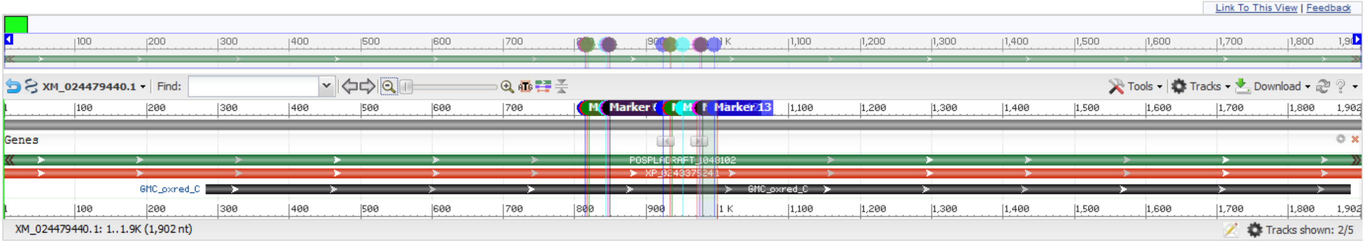

hypothetical protein POSPLADRAFT_1048102 [Postia placenta MAD-698-R-SB12]

NCBI Reference Sequence: XP_024337524.1

GenPept Identical Proteins FASTA

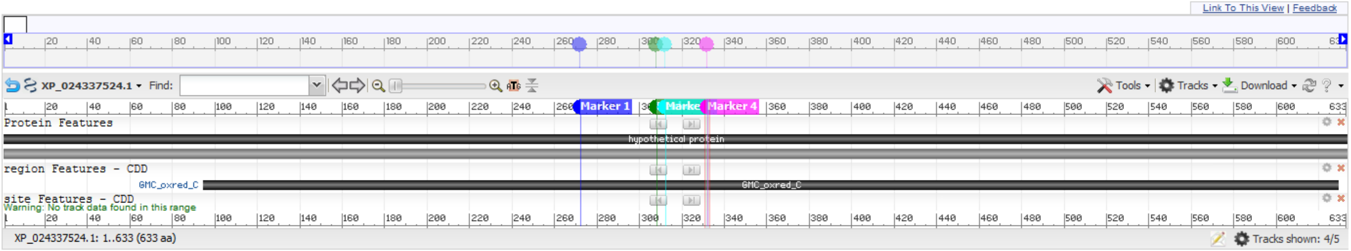

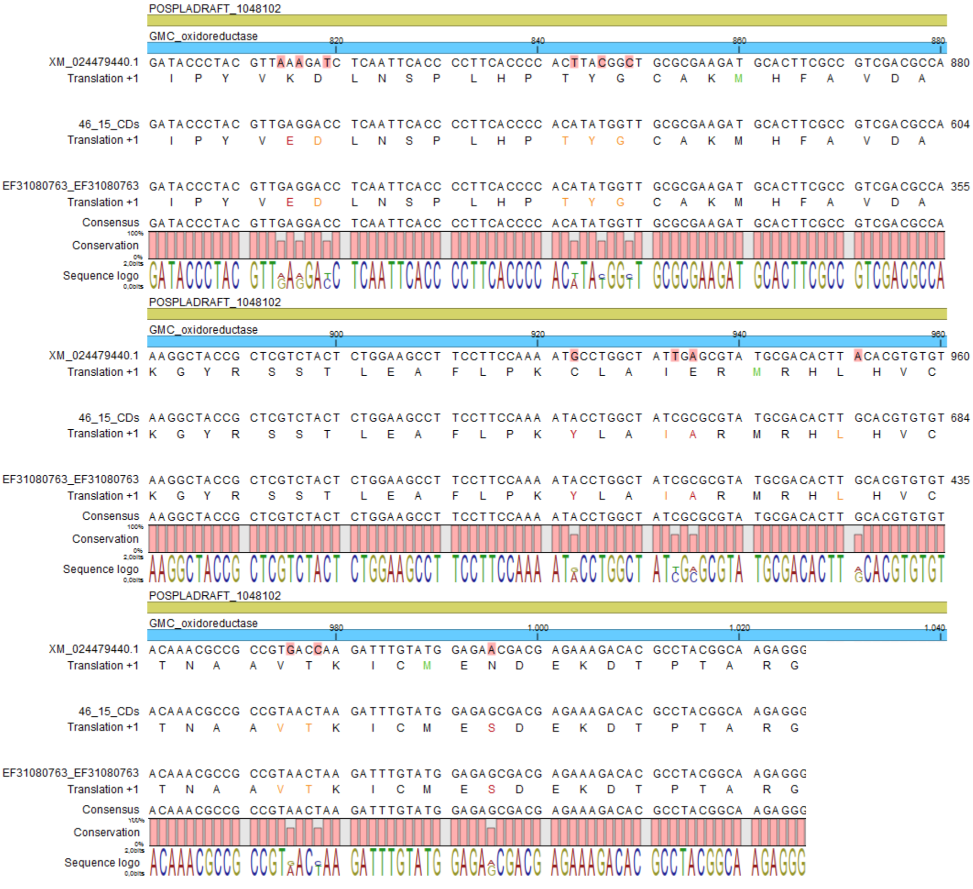

POSPLADRAFT_1133035
FPRL 280 _10_18
GH16 - POINT MUTATION POSITION 795 CDS

Postia placenta MAD-698-R-SB12 glycoside hydrolase family 16 protein (POSPLADRAFT_1133035), partial mRNA

NCBI Reference Sequence: XM_024484293.1
GenBank FASTA

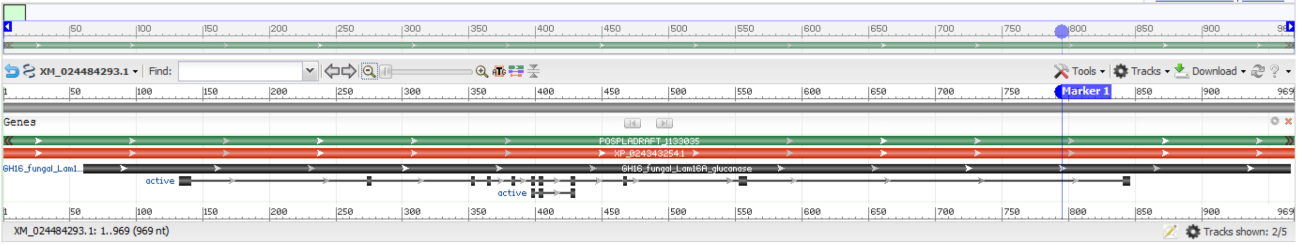


```
                                   POSPLADRAFT_1133035
                                   GH16_fungal_Lam16A_glucanase
XM_024484293              CAACAATCCC ACCTCCAATA GCTTCGGTCA CGACTTCAAC AATGCAGGTG GAGGTTGGTA TGCTATGGAG AGGACAAGCG ACGAAGTGAA 662
Translation ORF/CDS        N  N  P   T  S  N   S  F  G  H   D  F  N   N  A  G   G  G  W  Y   A  M  E   R  T  S   D  E  V  K

10_18 selection_CDS       CAACAATCCC ACCTCCAATA GCTTCGGTCA CGACTTCAAC AATGCAGGTG GAGGTTGGTA TGCTATGGAG AGGACAAGCG ACGAAGTGAA 647
Translation ORF/CDS        N  N  P   T  S  N   S  F  G  H   D  F  N   N  A  G   G  G  W  Y   A  M  E   R  T  S   D  E  V  K

2_EF31080772_EF31080772   CAACAATCCC ACCTCCAATA GCTTCGGTCA CGACTTCAAC AATGCAGGTG GAGGTTGGTA TGCTATGGAG AGGACAAGCG ACGAAGTGAA 590
Translation ORF/CDS        N  N  P   T  S  N   S  F  G  H   D  F  N   N  A  G   G  G  W  Y   A  M  E   R  T  S   D  E  V  K
Consensus                 CAACAATCCC ACCTCCAATA GCTTCGGTCA CGACTTCAAC AATGCAGGTG GAGGTTGGTA TGCTATGGAG AGGACAAGCG ACGAAGTGAA
Conservation
Sequence logo             CAACAATCCC ACCTCCAATA GCTTCGGTCA CGACTTCAAC AATGCAGGTG GAGGTTGGTA TGCTATGGAG AGGACAAGCG ACGAAGTGAA
```

```
                                   POSPLADRAFT_1133035
                                   GH16_fungal_Lam16A_glucanase
XM_024484293              AGTTTGGTTC TGGTCAAGAC AGGACAGCAC TGTCCCTGGC GATGTGCAGA GTGGTGCTGA CGAGGTCAAC ACCAACAACT GGAATCAACC 752
Translation ORF/CDS        V  W  F   W  S  R   Q  D  S  T   V  P  G   D  V  Q   S  G  A  D   E  V  N   T  N  N   W  N  Q  P

10_18 selection_CDS       AGTTTGGTTC TGGTCAAGAC AGGACAGCAC TGTCCCTGGC GATGTGCAGA GTGGTGCTGA CGAGGTCAAC ACCAACAACT GGAATCAACC 737
Translation ORF/CDS        V  W  F   W  S  R   Q  D  S  T   V  P  G   D  V  Q   S  G  A  D   E  V  N   T  N  N   W  N  Q  P

2_EF31080772_EF31080772   AGTTTGGTTC TGGTCAAGAC AGGACAGCAC TGTCCCTGGC GATGTGCAGA GTGGTGCTGA CGAGGTCAAC ACCAACAACT GGAATCAACC 680
Translation ORF/CDS        V  W  F   W  S  R   Q  D  S  T   V  P  G   D  V  Q   S  G  A  D   E  V  N   T  N  N   W  N  Q  P
Consensus                 AGTTTGGTTC TGGTCAAGAC AGGACAGCAC TGTCCCTGGC GATGTGCAGA GTGGTGCTGA CGAGGTCAAC ACCAACAACT GGAATCAACC
Conservation
Sequence logo             AGTTTGGTTC TGGTCAAGAC AGGACAGCAC TGTCCCTGGC GATGTGCAGA GTGGTGCTGA CGAGGTCAAC ACCAACAACT GGAATCAACC
```

```
                                   POSPLADRAFT_1133035
                                   GH16_fungal_Lam16A_glucanase
XM_024484293              GGTCGCGTAC TTCCCGAGCA CGGACTGTGA CATCGGTAAT GAGTTTGGCA AAAACAACAA CCTTATCTTC GATCTCACTT 832
Translation ORF/CDS        V  A  Y   F  P  S   T  D  C  D   I  G  N   E  F  G   K  N  N  N   L  I  F   D  L  T

10_18 selection_CDS       GGTCGCGTAC TTCCCGAGCA CGGACTGTGA CATCGGTAAT GAATTTGGCA AAAACAACAA CCTTATCTTC GATCTCACTT 817
Translation ORF/CDS        V  A  Y   F  P  S   T  D  C  D   I  G  N   E  F  G   K  N  N  N   L  I  F   D  L  T

2_EF31080772_EF31080772   GGTCGCGTAC TTCCCGAGCA CGGACTGTGA CATCGGTAAT GAATTTGGCA AAAACAACAA CCTTATCTTC GATCTCACTT 760
Translation ORF/CDS        V  A  Y   F  P  S   T  D  C  D   I  G  N   E  F  G   K  N  N  N   L  I  F   D  L  T
Consensus                 GGTCGCGTAC TTCCCGAGCA CGGACTGTGA CATCGGTAAT GAATTTGGCA AAAACAACAA CCTTATCTTC GATCTCACTT
Conservation
Sequence logo             GGTCGCGTAC TTCCCGAGCA CGGACTGTGA CATCGGTAAT GA TTTGGCA AAAACAACAA CCTTATCTTC GATCTCACTT
```

POSPLADRAFT_1146835

FPRL 280 _656_1

CBM18-GH16 –POINT MUTATION 549, 561, 564, 588, 615, 613, 699, 708, 735 CDs

Postia placenta MAD-698-R-SB12 glycoside hydrolase family 16 protein (POSPLADRAFT_1146835), partial mRNA

NCBI Reference Sequence: XM_024485992.1

GenBank FASTA

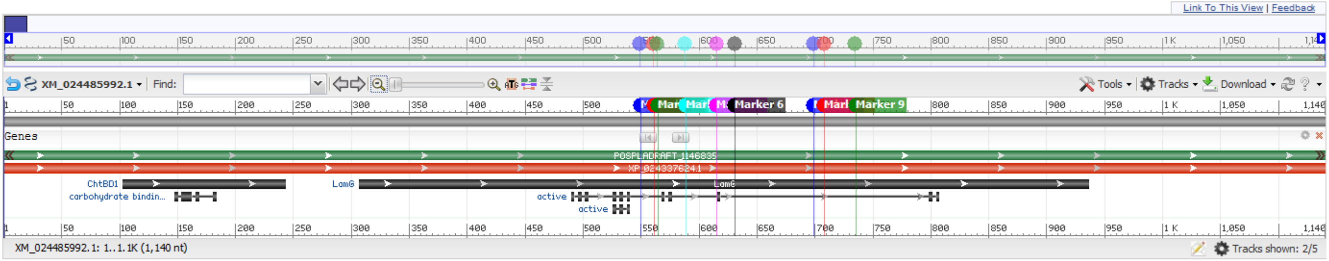


```
                               GH16 active site
         POSPLADRAFT_1146835
         LamG                          20                    40                    60                    80
XM_024485992.1         AAGACGGGCC GCTGGGGCGG TGTGGTGACG GCGTTCATCA CGATGTCGGA CATCAAGGAC GAAATCGACT GGGAGTTCCC 80
Translation +1           K  T  G   R  W  G  G   V  V  T   A  F  I   T  M  S  D   I  K  D   E  I  D   W  E  F  P

656_1 selection_CDS    AAGACGGGCC GCTGGGGCGG TGTGGTGACG GCGTTCATCA CGATGTCGGA CATCAAGGAC GAAATCGACT GGGAGTTCCC 80
Translation +1           K  T  G   R  W  G  G   V  V  T   A  F  I   T  M  S  D   I  K  D   E  I  D   W  E  F  P

EF31080767_EF31080767  AAGACGGGCC GCTGGGGCGG TGTGGTGACG GCGTTCATCA CGATGTCGGA CATCAAGGAC GAAATCGACT GGGAGTTCCC 80
Translation +1           K  T  G   R  W  G  G   V  V  T   A  F  I   T  M  S  D   I  K  D   E  I  D   W  E  F  P
Consensus              AAGACGGGCC GCTGGGGCGG TGTGGTGACG GCGTTCATCA CGATGTCGGA CATCAAGGAC GAAATCGACT GGGAGTTCCC
Conservation
Sequence logo
         GH16 active site
         POSPLADRAFT_1146835
         LamG                         100                   120                   140                   160
XM_024485992.1         CGGCAACGAG ACGTCCCAGG GCCAGACCAA CTACTT-CTG GCAGGGTGTC ATCC-CCGCA CAGACGGCCG GTCAGACGAC 158
Translation +1           G  N  E   T  S  Q   G  Q  T  N   Y  F  W   Q  G  V   I  P  A   Q  T  A   G  Q  T  T

656_1 selection_CDS    CGGTAACGAG ACGTCGCAAG GCCAGACCAA CTACTT-CTG GCAAGGTGTC ATCC-CCGCA CAGACGGCCG GCCAGACGAC 158
Translation +1           G  N  E   T  S  Q   G  Q  T  N   Y  F  W   Q  G  V   I  P  A   Q  T  A   G  Q  T  T

EF31080767_EF31080767  CGGTAACGAG ACGTCGCAAG GCCAGACCAA CTACTTTCTG GCAAGGTGTC ATCCGCCGCA CAGACGGCCG GCCAGACGAC 160
Translation +1           G  N  E   T  S  Q   G  Q  T  N   Y XXXX W   Q  G  V   I XXXX A   Q  T  A   G  Q  T  T
Consensus              CGGTAACGAG ACGTCGCAAG GCCAGACCAA CTACTT-CTG GCAAGGTGTC ATCC-CCGCA CAGACGGCCG GCCAGACGAC
Conservation
Sequence logo
         GH16 active site
         POSPLADRAFT_1146835
         LamG                         180                   200                   220                   240
XM_024485992.1         GGGCAACTTG ACGGACACGT TCAGCAACTA CCACGACTAC ACGATTGACT GGCAACCGGA CCAGCTGCAG TGGCTGGTCG 238
Translation +1           G  N  L   T  D  T   F  S  N  Y   H  D  Y   T  I  D   W  Q  P  D   Q  L  Q   W  L  V

656_1 selection_CDS    GGGCAACCTG ACGGACACGT TCAGCAACTA CCACGACTAC ACGATTGACT GGCAACCGGA CCAGCTGCAG TGGCTCGTCG 238
Translation +1           G  N  L   T  D  T   F  S  N  Y   H  D  Y   T  I  D   W  Q  P  D   Q  L  Q   W  L  V

EF31080767_EF31080767  GGGCAACCTG ACGGACACGT TCAGCAACTA CCACGACTAC ACGATTGACT GGCAACCGGA CCAGCTGCAG TGGCTCGTCG 240
Translation +1           G  N  L   T  D  T   F  S  N  Y   H  D  Y   T  I  D   W  Q  P  D   Q  L  Q   W  L  V

Consensus              GGGCAACCTG ACGGACACGT TCAGCAACTA CCACGACTAC ACGATTGACT GGCAACCGGA CCAGCTGCAG TGGCTCGTCG
Conservation
Sequence logo
         GH16 active site
         POSPLADRAFT_1146835
         LamG                         260                   280                   300                   320
XM_024485992.1         ACGGGAACGT GGTGCGCACG CTGCTGCGGT CGTCGGTGAC GGACAACAGC ACGGGCGTGA GCCGGTACCC GAACACGCCG 318
Translation +1         D  G  N  V   V  R  T   L  L  R   S  S  V  T   D  N  S   T  G  V   S  R  Y  P   N  T  P

656_1 selection_CDS    ACGGCAACGT GGTGCGCACG CTGCTGCGGT CATCGGTGAC GGACAACAGC ACGGGCGTGA GCCGGTACCC GAACACGCCG 318
Translation +1         D  G  N  V   V  R  T   L  L  R   S  S  V  T   D  N  S   T  G  V   S  R  Y  P   N  T  P

EF31080767_EF31080767  ACGGCAACGT GGTGCGCACG CTGCTGCGGT CATCGGTGAC GGACAACAGC ACGGGCGTGA GCCGGTACCC GAACACGCCG 320
Translation +1         D  G  N  V   V  R  T   L  L  R   S  S  V  T   D  N  S   T  G  V   S  R  Y  P   N  T  P
Consensus              ACGGCAACGT GGTGCGCACG CTGCTGCGGT CATCGGTGAC GGACAACAGC ACGGGCGTGA GCCGGTACCC GAACACGCCG
Conservation
Sequence logo
         GH16 active site
         POSPLADRAFT_1146835
         LamG
XM_024485992.1         AGCCGCATCG AGCTGAG 335
Translation +1           S  R  I   E  L

656_1 selection_CDS    AGCCGCATCG AGCTGAG 335
Translation +1           S  R  I   E  L

EF31080767_EF31080767  AGCCGCATCG AGCTGAG 337
Translation +1           S  R  I   E  L
Consensus              AGCCGCATCG AGCTGAG
Conservation
Sequence logo
```

POSPLADRAFT_1164613

FPRL 280 _14_15

GH5_5 – POINT MUTATION 222,225,243,252,330,333,348, 382,387,414,552,568,591,636,705,754, 759,780,798 CDs

AMINO ACID CHANGE POSITION 72, 126 PROTEIN

Postia placenta MAD-698-R-SB12 glycoside hydrolase family 5 protein (POSPLADRAFT_1164613), mRNA

NCBI Reference Sequence: XM_024487838.1

GenBank FASTA

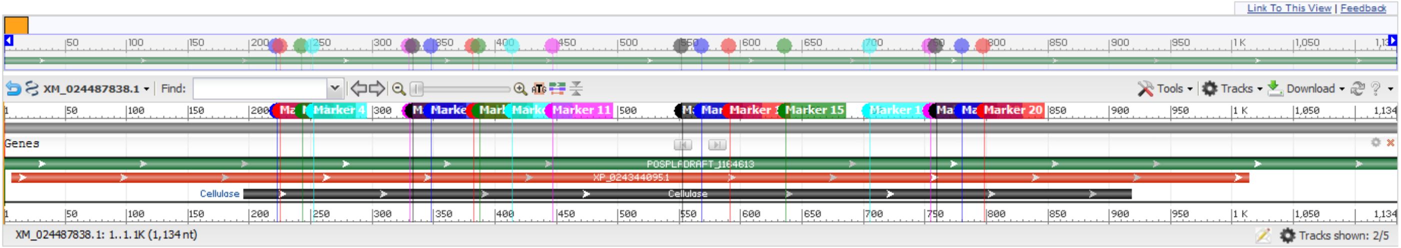

glycoside hydrolase family 5 protein [Postia placenta MAD-698-R-SB12]

NCBI Reference Sequence: XP_024344095.1

GenPept Identical Proteins FASTA

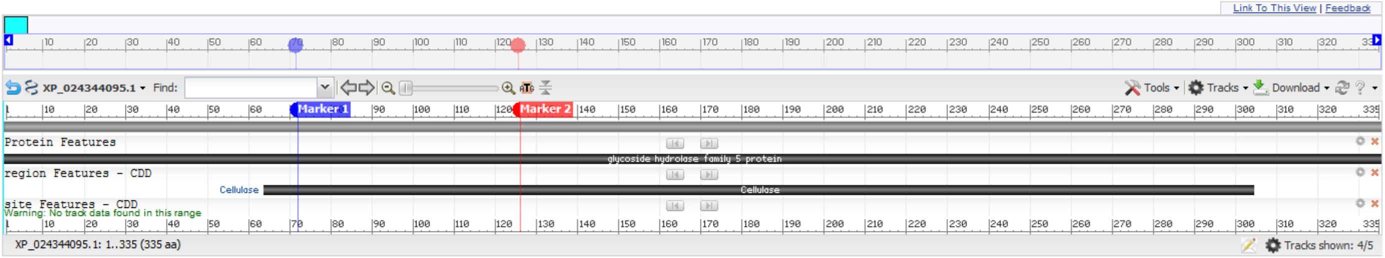

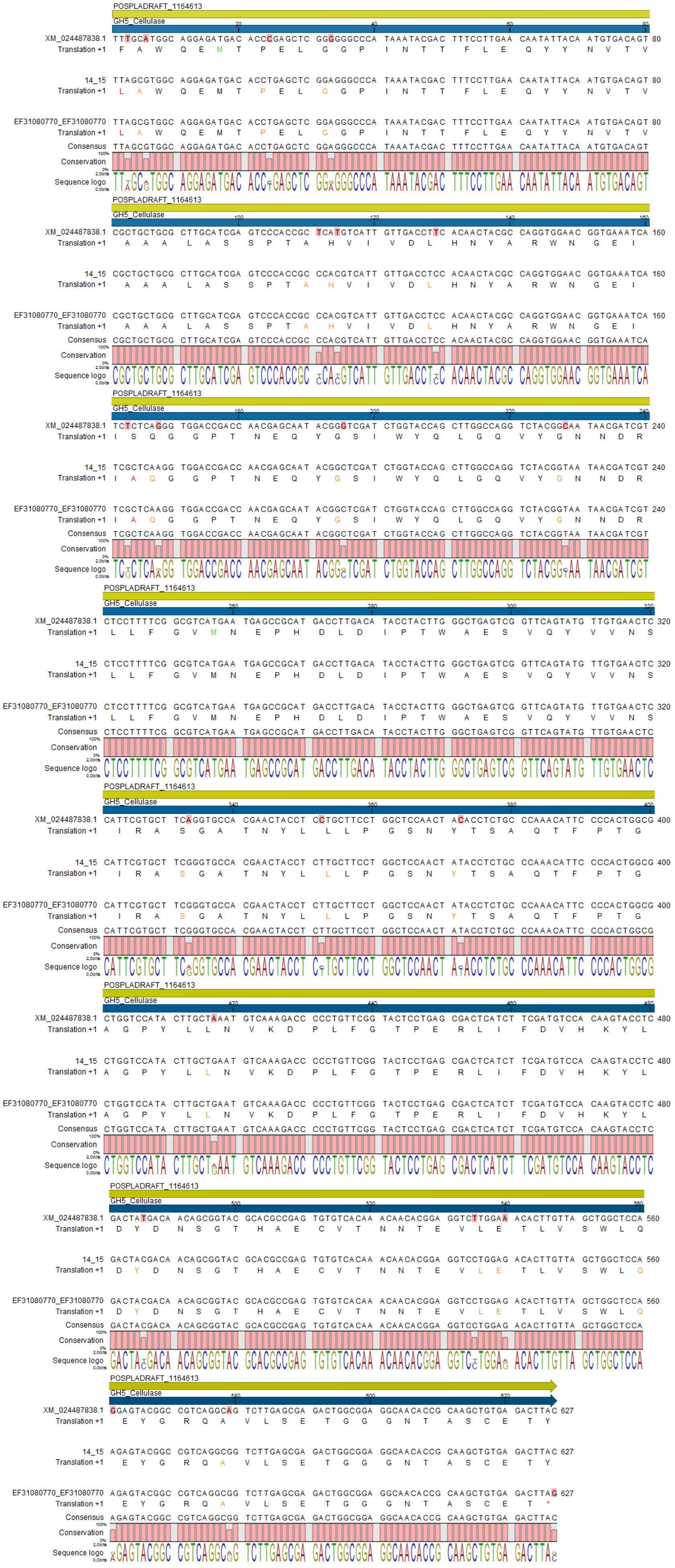

POSPLADRAFT_1164613
GH5_Cellulase 20 40 60 80
XM_024487838.1 TTTGCATGGC AGGAGATGAC ACCCGAGCTC GGGGGGCCCA TAAATACGAC TTTCCTTGAA CAATATTACA ATGTGACAGT 80
Translation +1 F A W Q E M T P E L G G P I N T T F L E Q Y Y N V T V
14_15 TTAGCGTGGC AGGAGATGAC ACCTGAGCTC GGAGGGCCCA TAAATACGAC TTTCCTTGAA CAATATTACA ATGTGACAGT 80
Translation +1 L A W Q E M T P E L G G P I N T T F L E Q Y Y N V T V
EF31080770_EF31080770 TTAGCGTGGC AGGAGATGAC ACCTGAGCTC GGAGGGCCCA TAAATACGAC TTTCCTTGAA CAATATTACA ATGTGACAGT 80
Translation +1 L A W Q E M T P E L G G P I N T T F L E Q Y Y N V T V
Consensus TTAGCGTGGC AGGAGATGAC ACCTGAGCTC GGAGGGCCCA TAAATACGAC TTTCCTTGAA CAATATTACA ATGTGACAGT
Conservation
Sequence logo
POSPLADRAFT_1164613
GH5_Cellulase 100 120 140 160
XM_024487838.1 CGCTGCTGCG CTTGCATCGA GTCCCACCGC TCATGTCATT GTTGACCTTC ACAACTACGC CAGGTGGAAC GGTGAAATCA 160
Translation +1 A A A L A S S P T A H V I V D L H N Y A R W N G E I
14_15 CGCTGCTGCG CTTGCATCGA GTCCCACCGC CCACGTCATT GTTGACCTCC ACAACTACGC CAGGTGGAAC GGTGAAATCA 160
Translation +1 A A A L A S S P T A H V I V D L H N Y A R W N G E I
EF31080770_EF31080770 CGCTGCTGCG CTTGCATCGA GTCCCACCGC CCACGTCATT GTTGACCTCC ACAACTACGC CAGGTGGAAC GGTGAAATCA 160
Translation +1 A A A L A S S P T A H V I V D L H N Y A R W N G E I
Consensus CGCTGCTGCG CTTGCATCGA GTCCCACCGC CCACGTCATT GTTGACCTCC ACAACTACGC CAGGTGGAAC GGTGAAATCA
Conservation
Sequence logo
POSPLADRAFT_1164613
GH5_Cellulase 180 200 220 240
XM_024487838.1 TCTCTCAGGG TGGACCGACC AACGAGCAAT ACGGGTCGAT CTGGTACCAG CTTGGCCAGG TCTACGGCAA TAACGATCGT 240
Translation +1 I S Q G G P T N E Q Y G S I W Y Q L G Q V Y G N N D R
14_15 TCGCTCAAGG TGGACCGACC AACGAGCAAT ACGGCTCGAT CTGGTACCAG CTTGGCCAGG TCTACGGTAA TAACGATCGT 240
Translation +1 I A Q G G P T N E Q Y G S I W Y Q L G Q V Y G N N D R
EF31080770_EF31080770 TCGCTCAAGG TGGACCGACC AACGAGCAAT ACGGCTCGAT CTGGTACCAG CTTGGCCAGG TCTACGGTAA TAACGATCGT 240
Translation +1 I A Q G G P T N E Q Y G S I W Y Q L G Q V Y G N N D R
Consensus TCGCTCAAGG TGGACCGACC AACGAGCAAT ACGGCTCGAT CTGGTACCAG CTTGGCCAGG TCTACGGTAA TAACGATCGT
Conservation
Sequence logo
POSPLADRAFT_1164613
GH5_Cellulase 260 280 300 320
XM_024487838.1 CTCCTTTTCG GCGTCATGAA TGAGCCGCAT GACCTTGACA TACCTACTTG GGCTGAGTCG GTTCAGTATG TTGTGAACTC 320
Translation +1 L L F G V M N E P H D L D I P T W A E S V Q Y V V N S
14_15 CTCCTTTTCG GCGTCATGAA TGAGCCGCAT GACCTTGACA TACCTACTTG GGCTGAGTCG GTTCAGTATG TTGTGAACTC 320
Translation +1 L L F G V M N E P H D L D I P T W A E S V Q Y V V N S
EF31080770_EF31080770 CTCCTTTTCG GCGTCATGAA TGAGCCGCAT GACCTTGACA TACCTACTTG GGCTGAGTCG GTTCAGTATG TTGTGAACTC 320
Translation +1 L L F G V M N E P H D L D I P T W A E S V Q Y V V N S
Consensus CTCCTTTTCG GCGTCATGAA TGAGCCGCAT GACCTTGACA TACCTACTTG GGCTGAGTCG GTTCAGTATG TTGTGAACTC
Conservation
Sequence logo
POSPLADRAFT_1164613
GH5_Cellulase 340 360 380 400
XM_024487838.1 CATTCGTGCT TCAGGTGCCA CGAACTACCT CCTGCTTCCT GGCTCCAACT ACACCTCTGC CCAAACATTC CCCACTGGCG 400
Translation +1 I R A S G A T N Y L L L P G S N Y T S A Q T F P T G
14_15 CATTCGTGCT TCGGGTGCCA CGAACTACCT CTTGCTTCCT GGCTCCAACT ATACCTCTGC CCAAACATTC CCCACTGGCG 400
Translation +1 I R A S G A T N Y L L L P G S N Y T S A Q T F P T G
EF31080770_EF31080770 CATTCGTGCT TCGGGTGCCA CGAACTACCT CTTGCTTCCT GGCTCCAACT ATACCTCTGC CCAAACATTC CCCACTGGCG 400
Translation +1 I R A S G A T N Y L L L P G S N Y T S A Q T F P T G
Consensus CATTCGTGCT TCGGGTGCCA CGAACTACCT CTTGCTTCCT GGCTCCAACT ATACCTCTGC CCAAACATTC CCCACTGGCG
Conservation
Sequence logo
POSPLADRAFT_1164613
GH5_Cellulase 420 440 460 480
XM_024487838.1 CTGGTCCATA CTTGCTAAAT GTCAAAGACC CCCTGTTCGG TACTCCTGAG CGACTCATCT TCGATGTCCA CAAGTACCTC 480
Translation +1 A G P Y L L N V K D P L F G T P E R L I F D V H K Y L
14_15 CTGGTCCATA CTTGCTGAAT GTCAAAGACC CCCTGTTCGG TACTCCTGAG CGACTCATCT TCGATGTCCA CAAGTACCTC 480
Translation +1 A G P Y L L N V K D P L F G T P E R L I F D V H K Y L
EF31080770_EF31080770 CTGGTCCATA CTTGCTGAAT GTCAAAGACC CCCTGTTCGG TACTCCTGAG CGACTCATCT TCGATGTCCA CAAGTACCTC 480
Translation +1 A G P Y L L N V K D P L F G T P E R L I F D V H K Y L
Consensus CTGGTCCATA CTTGCTGAAT GTCAAAGACC CCCTGTTCGG TACTCCTGAG CGACTCATCT TCGATGTCCA CAAGTACCTC
Conservation
Sequence logo
POSPLADRAFT_1164613
GH5_Cellulase 500 520 540 560
XM_024487838.1 GACTATGACA ACAGCGGTAC GCACGCCGAG TGTGTCACAA ACAACACGGA GGTCTTGGAA ACACTTGTTA GCTGGCTCCA 560
Translation +1 D Y D N S G T H A E C V T N N T E V L E T L V S W L Q
14_15 GACTACGACA ACAGCGGTAC GCACGCCGAG TGTGTCACAA ACAACACGGA GGTCCTGGAG ACACTTGTTA GCTGGCTCCA 560
Translation +1 D Y D N S G T H A E C V T N N T E V L E T L V S W L Q
EF31080770_EF31080770 GACTACGACA ACAGCGGTAC GCACGCCGAG TGTGTCACAA ACAACACGGA GGTCCTGGAG ACACTTGTTA GCTGGCTCCA 560
Translation +1 D Y D N S G T H A E C V T N N T E V L E T L V S W L Q
Consensus GACTACGACA ACAGCGGTAC GCACGCCGAG TGTGTCACAA ACAACACGGA GGTCCTGGAG ACACTTGTTA GCTGGCTCCA
Conservation
Sequence logo
POSPLADRAFT_1164613
GH5_Cellulase 580 600 620
XM_024487838.1 GGAGTACGGC CGTCAGGCAG TCTTGAGCGA GACTGGCGGA GGCAACACCG CAAGCTGTGA GACTTAC 627
Translation +1 E Y G R Q A V L S E T G G G N T A S C E T Y
14_15 AGAGTACGGC CGTCAGGCGG TCTTGAGCGA GACTGGCGGA GGCAACACCG CAAGCTGTGA GACTTAC 627
Translation +1 E Y G R Q A V L S E T G G G N T A S C E T Y
EF31080770_EF31080770 AGAGTACGGC CGTCAGGCGG TCTTGAGCGA GACTGGCGGA GGCAACACCG CAAGCTGTGA GACTTAG 627
Translation +1 E Y G R Q A V L S E T G G G N T A S C E T *
Consensus AGAGTACGGC CGTCAGGCGG TCTTGAGCGA GACTGGCGGA GGCAACACCG CAAGCTGTGA GACTTAC
Conservation
Sequence logo

POSPLADRAFT_1065808

FPRL 280 _327_2

CE15 – MANY POINT MUTATION CDs/ PROTEIN

21 pb Insertion after position 709 CDs

10 pb Deletion on FPRL_280 (position 773-782 Pospladraft)

Postia placenta MAD-698-R-SB12 carbohydrate esterase family 15 protein (POSPLADRAFT_1065808), partial mRNA

NCBI Reference Sequence: XM_024481997.1

GenBank FASTA

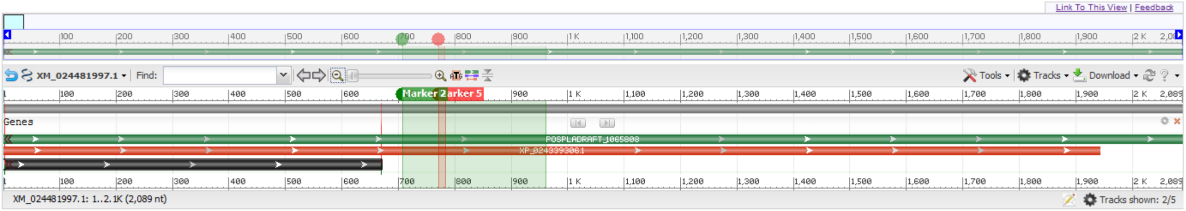


```
                      760                 780             800             820
XM_024481997.1        GATCTGA--- ---------- ---------CT GATGCGTGCA GCCGGCCAAA GCGGCGTCCA GGAGCCGAGG CTCATGGCCA GTCTATACTG 69
Translation +1         D  L                         T   D  A  C   S  R  P  K   R  R  P   G  A  E   A  H  G  Q   S  I  L

327_2 selection       GCCATCAGCC CAGACATATT CATCGGTTCT GATGCGTGCA GGCCGTCAAA GCGGCGTTCA GGAGCCAAGG CTCATAGCCA GCCTATTCTG 90
Translation +1         A  I  S   P  D  I  F   I  G  S   D  A  C   R  P  S  K   R  R  S   G  A  K   A  H  S  Q   P  I  L

EF31080773_EF31080773 GCCATCAGCC CAGACATATT CATCGGTTCT GATGCGTGCA GGCCGTCAAA GCGGCGTTCA GGAGCCAAGG CTCATAGCCA GCCTATTCTG 90
Translation +1         A  I  S   P  D  I  F   I  G  S   D  A  C   R  P  S  K   R  R  S   G  A  K   A  H  S  Q   P  I  L
Consensus             GCCATCAGCC CAGACATATT CATCGGTTCT GATGCGTGCA GGCCGTCAAA GCGGCGTTCA GGAGCCAAGG CTCATAGCCA GCCTATTCTG
Conservation

                      840                 860                 880             900             920
XM_024481997.1        TCTCTGAAAC TTCTCCGCTG CAATAATCTA CACGATCTGC TGCTGGCTAT GGCTGGACTG GGGCTTACCA TTTGCTCACT GTCCGGGGAT 159
Translation +1         S  L  K   L  L  R  C   N  N  L   H  D  L   L  L  A  M   A  G  L   G  L  T   I  C  S  L   S  G  D

327_2 selection       T--------- -TCTCCGCTG CAAAAGCCCA CACGCTCTGC GTCTGGATAC GGCTGGACTG CGGCTTACCA TTTGCTCACT GTCCGGAGAT 170
Translation +1                    F  S  A   A  K  A  H   T  L  C   V  W  I   R  L  D  C   G  L  P   F  A  H   C  P  E  I

EF31080773_EF31080773 T--------- -TCTCCGCTG CAAAAGCCCA CACGCTCTGC GTCTGGATAC GGCTGGACTG CGGCTTACCA TTTGCTCACT GTCCGGAGAT 170
Translation +1                    F  S  A   A  K  A  H   T  L  C   V  W  I   R  L  D  C   G  L  P   F  A  H   C  P  E  I
Consensus             T--------- -TCTCCGCTG CAAAAGCCCA CACGCTCTGC GTCTGGATAC GGCTGGACTG CGGCTTACCA TTTGCTCACT GTCCGGAGAT
Conservation

                                  940                 960             980             1.000
XM_024481997.1        TATGCGGGT- ACCAATTACC ATGCGACTCT TGAACGCGCC TTCATTAAGA GAGACGGCAG AACCGCGGAA ATCGCAGCTT CCGGTAGCCT 248
Translation +1         Y  A  G    T  N  Y   H  A  T  L   E  R  A   F  I  K   R  D  G  R   T  A  E   I  A  A   S  G  S  L

327_2 selection       ACCGCGGGTC GCAAACTATC ATGCGACTTT TGAACACGCC TTCATGAAGA TAAACGGCAG AATCGCGGAA ATTGCAACTT CCGGTAGTCT 260
Translation +1          P  R  V   A  N  Y   H  A  T  F   E  H  A   F  M  K   I  N  G  R   I  A  E   I  A  T   S  G  S  L

EF31080773_EF31080773 ACCGCGGGTC GCAAACTATC ATGCGACTTT TGAACACGCC TTCATGAAGA TAAACGGCAG AATCGCGGAA ATTGCAACTT CCGGTAGTCT 260
Translation +1          P  R  V   A  N  Y   H  A  T  F   E  H  A   F  M  K   I  N  G  R   I  A  E   I  A  T   S  G  S  L
Consensus             ACCGCGGGTC GCAAACTATC ATGCGACTTT TGAACACGCC TTCATGAAGA TAAACGGCAG AATCGCGGAA ATTGCAACTT CCGGTAGTCT
Conservation

                      1.020
XM_024481997.1        TGTGAT 254
Translation +1          V

327_2 selection       TGTGAT 266
Translation +1          V

EF31080773_EF31080773 TGTGAT 266
Translation +1          V
Consensus             TGTGAT
Conservation
```
