# Supplementary material for: Degradative Capacity of Two Strains of Rhodonia placenta: From Phenotype to Genotype
Source: Front Microbiol. 2020 Jun 18;11:1338. doi: 10.3389/fmicb.2020.01338 (PMC7314958; doi:10.3389/fmicb.2020.01338)
Supplement: Supplementary file 5 [file Data_Sheet_5.pdf]

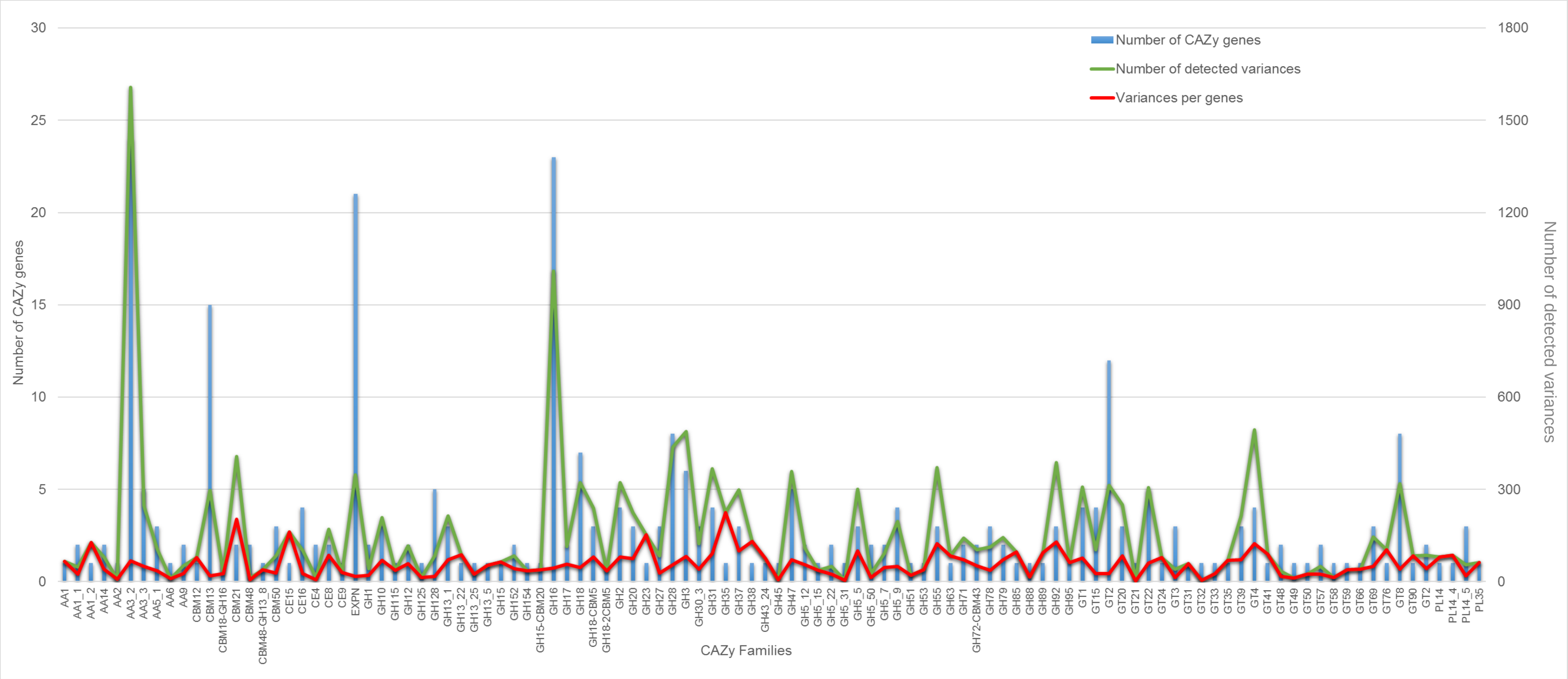

Supplementary Figure 1
Result of the variant analysis of all CAZy genes of FPRL280 compared to MAD-SB12 (predicted CDS only); Shown is the number of CAZy genes belonging to each subfamily of the *R. placenta* genomes (blue bars), all variants occuring in each subfamily (green line) as well as normalized variances, according to the gene size of each CAZy family.

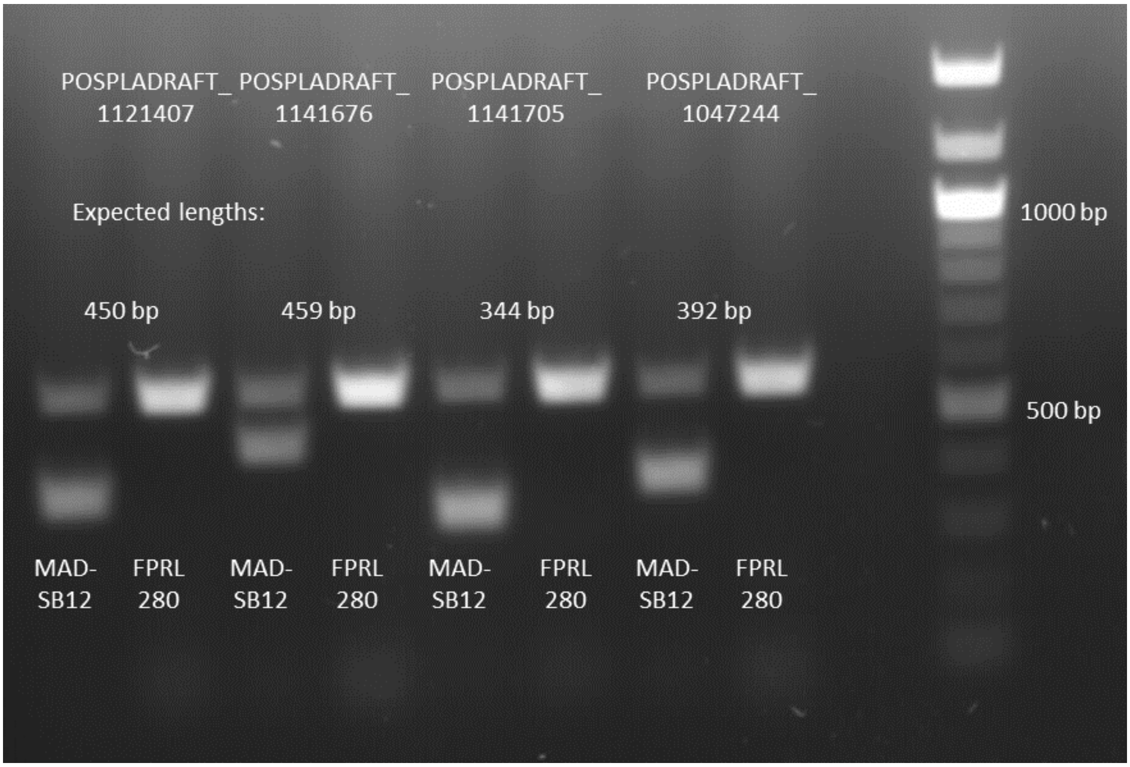

Supplementary Figure 2
PCR-based verification of the absence of four AA3_2 genes in the genome of FPRL280 vs. MAD-SB12.
Template in lines 1, 3, 5 and 7 was MAD-SB12 gDNA; Template in lines 2, 4, 6 and 8 was FPRL280 gDNA. The expected size of the respective amplicons is indicated above the observed bands. The control amplification of a common gene (POSPLADRAFT_1069652, a GH3) used as positive control in each reaction had an expected size of 562 bp.

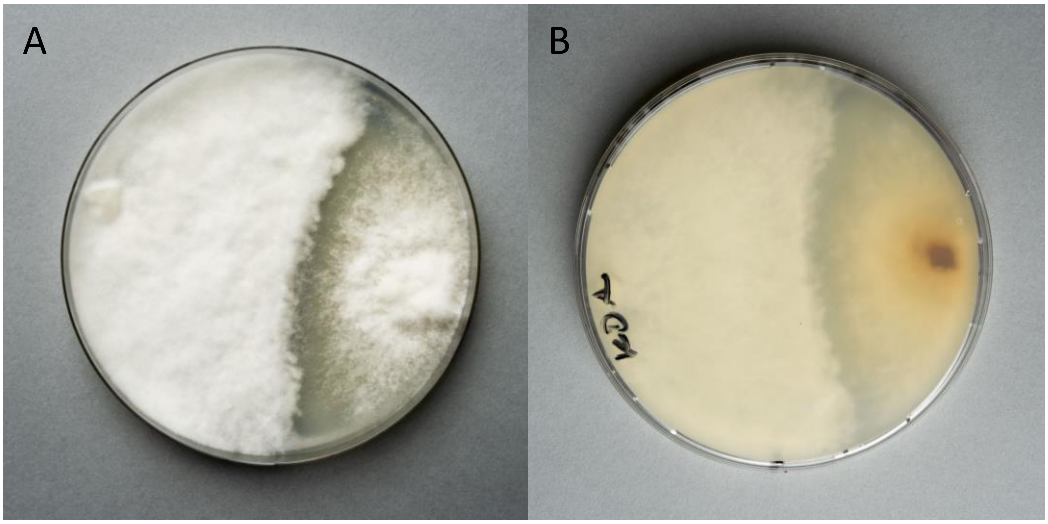

Supplementary Figure 3
Fusion test with the two monokaryotic *Rhodonia placenta* strains FPRL280 (left side) and MAD-SB12 (right side), showing that they are not able to fuse and further seem to form a barrier which is not overgrown.

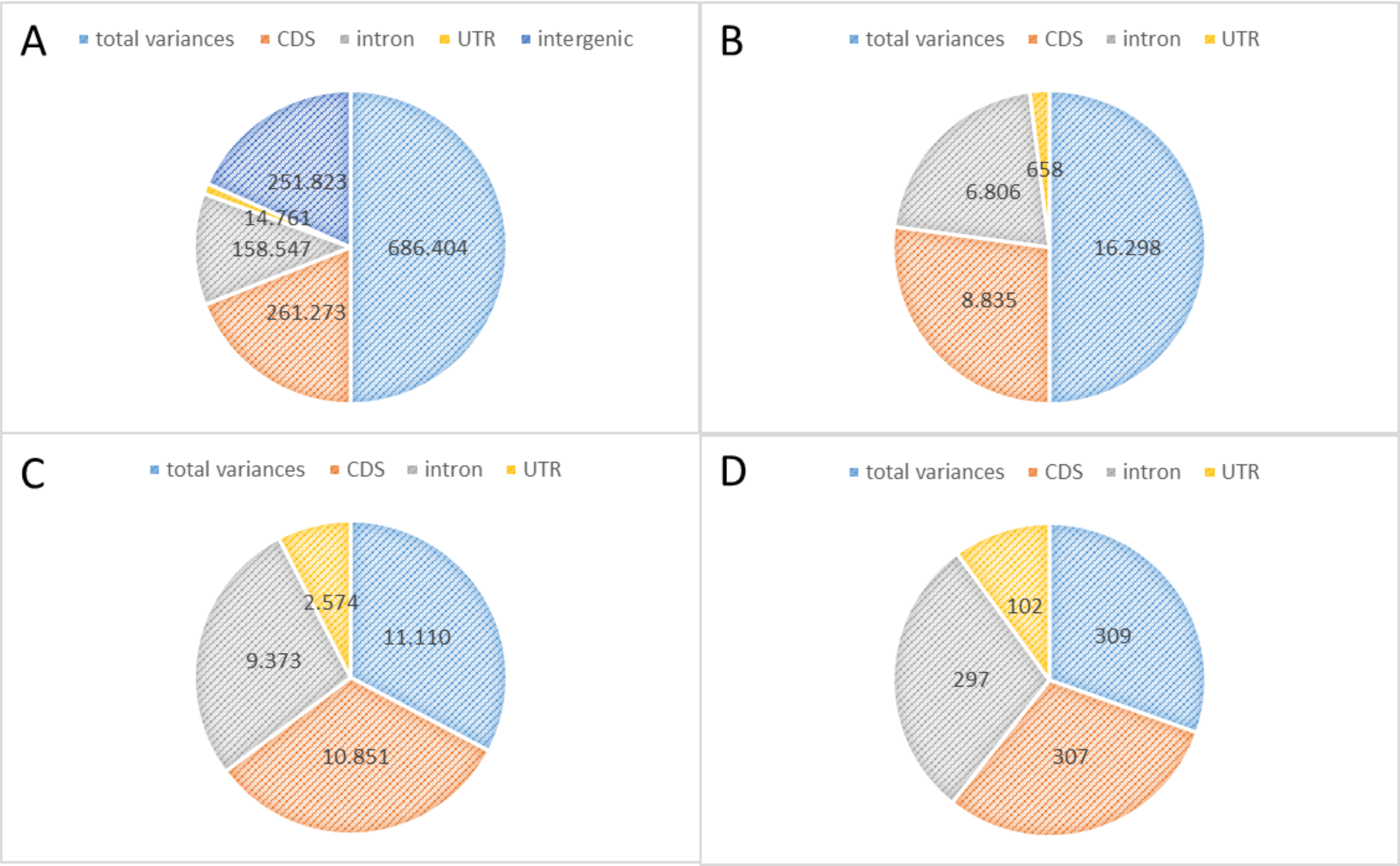

Supplementary Figure 4
Distribution of SNPs over the gene regions. (A) Total occurrences of variances in all genes and their distribution over the different gene regions. (B) All occurrences in CAZy genes. (C) Number of genes affected through variants. (D) Number of predicted CAZy genes which are affected.
