# Supplementary material for: Degradative Capacity of Two Strains of Rhodonia placenta: From Phenotype to Genotype
Source: Front Microbiol. 2020 Jun 18;11:1338. doi: 10.3389/fmicb.2020.01338 (PMC7314958; doi:10.3389/fmicb.2020.01338)
Supplement: Supplementary file 6 [file Table_1.PDF]

Primer List

MAD-SB12_ID POSPLADRAFT_	FPRL_280_ID	CAZy Family	Primer	F-Forward R-Reverse
**1050820**	88_4	GH31	ACTCGACATTGACGGTGCAT	F
			GCTAACAAGTGTGCTAGCGG	R
**1164613**	14_15	GH5	CGCTTTCTGCAGCTTTTGGT	F
			GCCGAGCACTCCGTCAATA	R
**1048102**	46_15	AA3_2	AGCTTGTGTGAGGGATATGCA	F
			GAGGTTAAGGGTGGCGTTCA	R
**1146835**	656_1	CBM18-GH16	GGCAACATCCAGAACACGTC	F
			CCAATGTCGCAAACACGCC	R
**1133035**	10_18	GH16	CGCCTTCAGAGCCAGGATAG	F
			AGTCTCCGCCTGTTTCCAAG	R
**1044277**	3_68	CBM13	TCTTCTTCAACAAAGGCGTATTCA	F
			CGGTGATCCTAAAATCCGTGCG	R
**1065808**	327_3	CE15	CATTTATGTGGCGCTGGACG	F
			AACCGACTCACGGGGATGAT	R
**1069652**	9_74	GH3	GCCCACTCACCACCAATACT	F
			GGCCTGTTCGAGAACCTCAG	R
**1121407**	-	AA3	TCCTTCAGGAAGTGACATCTCA	F
			AGTAGAAGGAATTATAGGACGCCT	R
**1141676**	-	AA3	CGCGAGTTTGGTATGACAGC	F
			AGCCATGATACTGAGCAGCG	R
**1141705**	-	AA3	CAATATGGCTGAACGTGGCA	F
			TTGACCCTCGCACAGGAAC	R
**1047244**	-	AA3	CTGGTACTCGAGGCAGGAAG	F
			TCCCACTTTCTCGACAGCTT	R
